# Supplementary material for: Development of a Library of Disulfide Bond-Containing Cationic Lipids for mRNA Delivery
Source: Pharmaceutics. 2023 Feb 1;15(2):477. doi: 10.3390/pharmaceutics15020477 (PMC9961079; doi:10.3390/pharmaceutics15020477)
Supplement: Supplementary file 1 [file pharmaceutics-15-00477-s001.zip › pharmaceutics-2173947-supplementary.pdf]

pharmaceutics

MDPI

# Supplementary Materials: Development of a Library of Disulfide Bond-containing Cationic Lipids for mRNA Delivery

**Zhigao Shen, Cong Liu, Ziqian Wang, Fengfei Xie, Xingwu Liu, Lingkai Dong, Xuehua Pan, Chen Zeng and Peng George Wang**

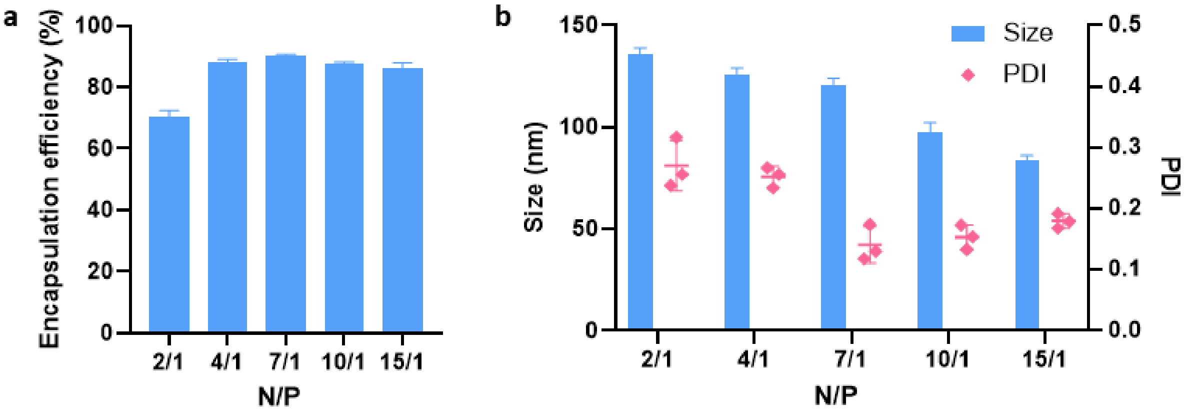


**Figure S1.** Characterization of LNP with different N/P ratio using C4S18A formulation. **a**) encapsulation efficiency of LNP with different N/P ratio. **b**) the variance of particle size and PDI over N/P ratio. Data presented as mean ± SEM of three separate experiments.

### Characterization of disulfide lipids

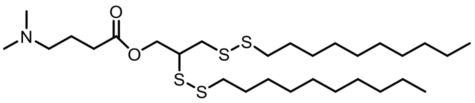


**2,3-bis(decyldisulfanyl)propyl 4-(dimethylamino)butanoate (C4S10)**

Synthesized by using the general procedure. $^1$H NMR (500 MHz, $CDCl_3$) $\delta$ = 4.39 – 4.33 (m, 2H), 3.30 – 3.25 (m, 1H), 3.05 (dd, $J$ = 13.8, 6.2 Hz, 1H), 2.92 (dd, $J$ = 13.8, 8.1 Hz, 1H), 2.72 – 2.68 (m, 4H), 2.38 (t, $J$ = 7.5 Hz, 2H), 2.30 (t, $J$ = 7.1 Hz, 2H), 2.22 (s, 6H), 1.84 – 1.77 (m, 2H), 1.71 – 1.63 (m, 4H), 1.41 – 1.35 (m, 4H), 1.32 – 1.24 (m, 24H), 0.88 (t, $J$ = 7.0 Hz, 6H). $^{13}$C NMR (101 MHz, $CDCl_3$) $\delta$ = 173.30, 64.14, 58.86, 49.26, 45.49, 40.19, 39.96, 39.03, 32.06, 32.04, 29.70, 29.67, 29.66, 29.46, 29.38, 29.38, 29.34, 29.31, 28.66, 28.64, 22.94, 22.82, 14.27. **HR-MS** (ESI) *m/z* calcd for $C_{29}H_{60}O_2NS_4$ $[M+H]^+$ 582.3501, found 582.3503.

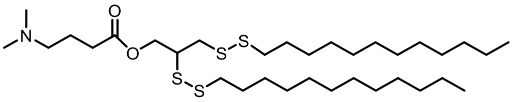


**2,3-bis(dodecyldisulfanyl)propyl 4-(dimethylamino)butanoate (C4S12)**

Synthesized by using the general procedure. $^1$H NMR (400 MHz, $CDCl_3$) $\delta$ = 4.36 (d, $J$ = 5.7 Hz, 2H), 3.28 (dq, $J$ = 8.1, 5.8 Hz, 1H), 3.08 – 3.02 (m, 1H), 2.91 (dd, $J$ = 13.8, 8.2 Hz, 1H), 2.74 – 2.67 (m, 4H), 2.41 (dd, $J$ = 15.5, 8.1 Hz, 4H), 2.31 (s, 6H), 1.91 – 1.82 (m, 2H), 1.71 – 1.62 (m, 4H), 1.41 – 1.34 (m, 4H), 1.31 – 1.24 (m, 32H), 0.87 (t, $J$ = 6.8 Hz, 6H). $^{13}$C NMR (101 MHz, $CDCl_3$) $\delta$ = 173.07, 64.20, 58.58, 49.22, 45.08, 40.15, 39.98, 39.03, 32.06, 31.86, 29.81, 29.79, 29.76, 29.68, 29.67, 29.50, 29.39, 29.35, 29.32, 28.66, 28.64, 22.84, 22.41, 14.28. **HR-MS** (ESI) *m/z* calcd for $C_{33}H_{68}O_2NS_4$ $[M+H]^+$ 638.4127, found 638.4132.

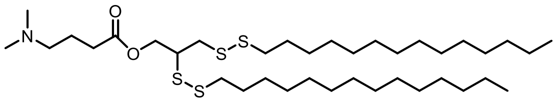

**2,3-bis(tetradecyldisulfanyl)propyl 4-(dimethylamino)butanoate (C4S14)**

Synthesized by using the general procedure. $^{1}$H NMR (400 MHz, $CDCl_3$) $\delta$ = 4.39 – 4.33 (m, 2H), 3.28 (dq, $J$ = 8.0, 5.8 Hz, 1H), 3.05 (dd, $J$ = 13.7, 6.2 Hz, 1H), 2.95 – 2.88 (m, 1H), 2.70 (td, $J$ = 7.7, 2.3 Hz, 4H), 2.38 (t, $J$ = 7.5 Hz, 2H), 2.30 (d, $J$ = 7.5 Hz, 2H), 2.23 (s, 6H), 1.85 – 1.76 (m, 2H), 1.67 (dq, $J$ = 14.4, 7.1 Hz, 4H), 1.43 – 1.34 (m, 4H), 1.31 – 1.22 (m, 40H), 0.88 (t, $J$ = 6.8 Hz, 6H). $^{13}$C NMR (101 MHz, $CDCl_3$) $\delta$ = 173.30, 64.15, 58.85, 49.26, 45.46, 40.19, 39.97, 39.04, 32.07, 29.85, 29.84, 29.81, 29.77, 29.68, 29.68, 29.52, 29.40, 29.39, 29.35, 29.32, 28.67, 28.65, 22.91, 22.85, 14.28. **HR-MS** (ESI) *m/z* calcd for $C_{37}H_{76}O_2NS_4$ $[M+H]^+$ 694.4759, found 694.4763.

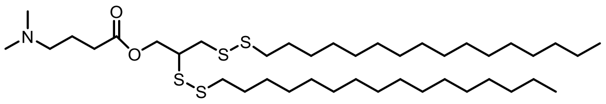

**2,3-bis(hexadecyldisulfanyl)propyl 4-(dimethylamino)butanoate (C4S16)**

Synthesized by using the general procedure. $^{1}$H NMR (400 MHz, $CDCl_3$) $\delta$ = 4.35 (dd, $J$ = 9.2, 3.8 Hz, 2H), 3.27 (dq, $J$ = 8.0, 5.8 Hz, 1H), 3.03 (dd, $J$ = 12.4, 6.2 Hz, 1H), 2.91 (dd, $J$ = 13.7, 8.1 Hz, 1H), 2.70 (td, $J$ = 7.8, 2.1 Hz, 4H), 2.42 – 2.32 (m, 4H), 2.26 (s, 6H), 1.82 – 1.77 (m, 2H), 1.71 – 1.62 (m, 4H), 1.39 – 1.22 (m, 52H), 0.87 (t, $J$ = 6.8 Hz, 6H). $^{13}$C NMR (101 MHz, $CDCl_3$) $\delta$ = 173.19, 64.16, 58.75, 49.24, 45.32, 40.17, 39.96, 39.03, 32.07, 31.97, 29.84, 29.81, 29.76, 29.68, 29.67, 29.51, 29.39, 29.39, 29.34, 29.31, 28.66, 28.64, 22.84, 22.72, 14.27. **HR-MS** (ESI) *m/z* calcd for $C_{41}H_{84}O_2NS_4$ $[M+H]^+$ 750.5379, found 750.5388

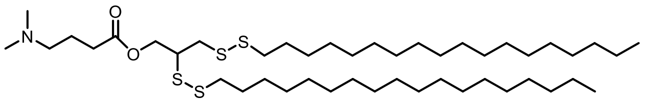

**2,3-bis(octadecyldisulfanyl)propyl 4-(dimethylamino)butanoate (C4S18)**

Synthesized by using the general procedure. $^{1}$H NMR (400 MHz, $CDCl_3$) $\delta$ = 4.35 (dd, $J$ = 5.6, 1.2 Hz, 2H), 3.27 (dq, $J$ = 8.0, 5.8 Hz, 1H), 3.04 (dd, $J$ = 13.7, 6.2 Hz, 1H), 2.91 (dd, $J$ = 13.8, 8.1 Hz, 1H), 2.69 (td, $J$ = 7.7, 2.1 Hz, 4H), 2.36 (dt, $J$ = 15.1, 7.5 Hz, 4H), 2.24 (s, 6H), 1.85 – 1.77 (m, 2H), 1.66 (dq, $J$ = 14.4, 7.1 Hz, 4H), 1.39 – 1.20 (m, 60H), 0.86 (t, $J$ = 6.8 Hz, 6H). $^{13}$C NMR (101 MHz, $CDCl_3$) $\delta$ = 173.17, 64.14, 58.68, 49.22, 45.25, 40.15, 39.94, 39.00, 32.05, 31.96, 29.82, 29.79, 29.74, 29.65, 29.65, 29.49, 29.37, 29.36, 29.32, 29.29, 28.64, 28.62, 22.81, 22.68, 14.25. **HR-MS** (ESI) *m/z* calcd for $C_{45}H_{92}O_2NS_4$ $[M+H]^+$ 806.6005, found 806.6009.

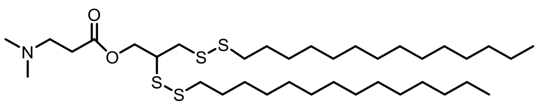

**2,3-bis(tetradecyldisulfanyl)propyl 3-(dimethylamino)propanoate (C3S14)**

Synthesized by using the general procedure. $^{1}$H NMR (500 MHz, $CDCl_3$) $\delta$ = 4.42 – 4.34 (m, 2H), 3.28 (dq, $J$ = 7.8, 5.9 Hz, 1H), 3.04 (dd, $J$ = 13.8, 6.3 Hz, 1H), 2.93 (dd, $J$ = 13.6, 8.3 Hz, 1H), 2.73 – 2.68 (m, 4H), 2.65 – 2.60 (m, 2H), 2.52 (dd, $J$ = 8.8, 5.3 Hz, 2H), 2.25 (s, 6H), 1.71 – 1.63 (m, 4H), 1.37 (d, $J$ = 4.8 Hz, 4H), 1.30 – 1.24 (m, 40H), 0.87 (t, $J$ = 6.9 Hz, 6H). $^{13}$C NMR (101 MHz, $CDCl_3$) $\delta$ = 172.19, 64.31, 54.75, 49.25, 45.34, 40.17, 39.97, 39.03, 32.88, 32.07, 29.84, 29.83, 29.81, 29.76, 29.68, 29.67, 29.51, 29.40, 29.39, 29.35, 29.32, 28.67, 28.64, 22.84, 14.28. **HR-MS** (ESI) *m/z* calcd for $C_{36}H_{74}O_2NS_4$ $[M+H]^+$ 680.4597, found 680.4601.

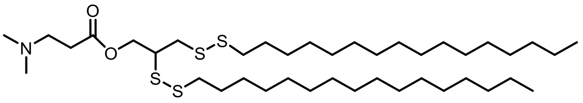

**2,3-bis(hexadecyldisulfanyl)propyl 3-(dimethylamino)propanoate (C3S16)**

Synthesized by using the general procedure. $^1$H NMR (500 MHz, $CDCl_3$) $\delta$ = 4.42 – 4.34 (m, 2H), 3.28 (dq, *J* = 7.8, 5.8 Hz, 1H), 3.05 (dd, *J* = 13.7, 6.3 Hz, 1H), 2.98 – 2.90 (m, 1H), 2.71 (t, *J* = 6.8 Hz, 4H), 2.63 (dd, *J* = 10.9, 3.9 Hz, 2H), 2.51 (dd, *J* = 8.8, 5.3 Hz, 2H), 2.24 (s, 6H), 1.71 – 1.63 (m, 4H), 1.40 – 1.35 (m, 4H), 1.30 – 1.23 (m, 48H), 0.88 (t, *J* = 7.0 Hz, 6H). $^{13}$C NMR (101 MHz, $CDCl_3$) $\delta$ = 172.21, 64.31, 54.79, 49.27, 45.38, 40.18, 39.98, 39.04, 32.94, 32.07, 29.85, 29.81, 29.77, 29.68, 29.52, 29.40, 29.36, 29.32, 28.67, 28.65, 22.84, 14.28. **HR-MS** (ESI) *m*/*z* calcd for $C_{40}H_{82}O_2NS_4$ $[M+H]^+$ 736.5223, found 736.5231.

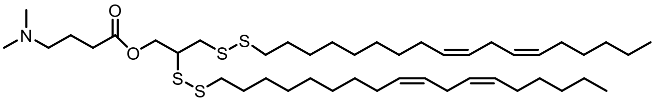

**2,3-bis(((9Z,12Z)-octadeca-9,12-dien-1-yl)disulfanyl)propyl 4-(dimethylamino) butanoate (C4S18A)**

Synthesized by using the general procedure. $^1$H NMR (400 MHz, $CDCl_3$) $\delta$ = 5.42 – 5.29 (m, 8H), 4.36 (d, *J* = 5.6 Hz, 2H), 3.28 (dq, *J* = 11.8, 5.8 Hz, 1H), 3.05 (dd, *J* = 13.7, 6.2 Hz, 1H), 2.92 (dt, *J* = 12.6, 3.7 Hz, 1H), 2.77 (t, *J* = 6.4 Hz, 4H), 2.70 (t, *J* = 6.6 Hz, 4H), 2.39 (t, *J* = 7.4 Hz, 2H), 2.34 (t, *J* = 7.4 Hz, 2H), 2.25 (s, 6H), 2.05 (dd, *J* = 13.7, 6.8 Hz, 8H), 1.86 – 1.77 (m, 2H), 1.72 – 1.62 (m, 4H), 1.42 – 1.24 (m, 32H), 0.89 (t, *J* = 6.7 Hz, 6H). $^{13}$C NMR (101 MHz, $CDCl_3$) $\delta$ = 173.24, 130.36, 130.21, 128.17, 128.05, 64.17, 58.79, 49.27, 45.37, 40.20, 39.97, 39.00, 32.01, 31.67, 29.80, 29.57, 29.56, 29.49, 29.39, 29.36, 29.36, 29.33, 29.30, 28.65, 28.62, 27.36, 27.35, 25.77, 22.80, 22.72, 14.23. **HR-MS** (ESI) *m*/*z* calcd for $C_{45}H_{84}O_2NS_4$ $[M+H]^+$ 798.5379, found 798.5364.

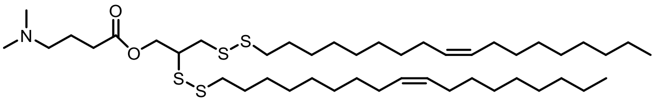

**2,3-bis(((Z)-octadec-9-en-1-yl)disulfanyl)propyl 4-(dimethylamino)butanoate (C4S18B)**

Synthesized by using the general procedure. $^1$H NMR (500 MHz, $CDCl_3$) $\delta$ = 5.37 – 5.30 (m, 4H), 4.36 (dd, *J* = 5.6, 2.0 Hz, 2H), 3.27 (dq, *J* = 7.8, 5.8 Hz, 1H), 3.04 (dd, *J* = 13.7, 6.2 Hz, 1H), 2.91 (dd, *J* = 13.8, 8.1 Hz, 1H), 2.70 (td, *J* = 7.7, 2.1 Hz, 4H), 2.36 (dd, *J* = 15.8, 7.6 Hz, 4H), 2.26 (s, 6H), 2.03 – 1.96 (m, 8H), 1.70 – 1.63 (m, 2H), 1.70 – 1.62 (m, 4H), 1.38 – 1.23 (m, 44H), 0.87 (t, *J* = 6.9 Hz, 6H). $^{13}$C NMR (101 MHz, $CDCl_3$) $\delta$ = 173.17, 130.10, 129.88, 64.15, 58.69, 49.21, 45.25, 40.14, 39.93, 38.97, 32.03, 31.94, 29.89, 29.87, 29.83, 29.79, 29.74, 29.65, 29.55, 29.45, 29.37, 29.35, 29.34, 29.31, 29.28, 28.63, 28.61, 27.34, 27.31, 22.82, 22.66, 14.26. **HR-MS** (ESI) *m*/*z* calcd for $C_{45}H_{88}O_2NS_4$ $[M+H]^+$ 802.5692, found 802.5699.

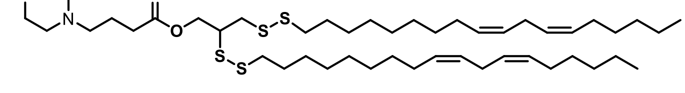

**2,3-bis(((9Z,12Z)-octadeca-9,12-dien-1-yl)disulfanyl)propyl 4-(4-methylpiperazin - 1-yl)butanoate (C4AS18A)**

$^1$H NMR (400 MHz, $CDCl_3$) $\delta$ 5.39 – 5.30 (m, 8H), 4.35 (d, *J* = 5.6 Hz, 2H), 3.27 (dq, *J* = 8.3, 5.8 Hz, 1H), 3.04 (dd, *J* = 13.7, 6.2 Hz, 1H), 2.95 – 2.88 (m, 1H), 2.76 (t, *J* = 6.5 Hz, 4H), 2.69 (dt, *J* = 7.2, 3.7 Hz, 4H), 2.36 (t, *J* = 7.3 Hz, 6H), 2.28 (s, 3H), 2.03 (q, *J* = 6.9 Hz, 8H), 1.82 (q, *J* = 7.3 Hz, 2H), 1.66 (q, *J* = 7.7 Hz, 4H), 1.39 – 1.14 (m, 34H), 0.87 (t, *J* = 6.7 Hz, 6H). $^{13}$C NMR (101 MHz, $CDCl_3$) $\delta$ 173.24, 130.31, 130.16, 128.12, 127.99, 64.10, 57.60, 55.16, 53.05, 49.19, 46.06, 40.13, 39.89, 38.93, 32.18, 31.64, 29.76, 29.54, 29.46, 29.36, 29.33, 29.30, 29.26,

28.62, 28.59, 27.32, 27.32, 27.31, 25.74, 22.70, 22.17, 14.23. **HR-MS** (ESI) *m/z* calcd for $C_{48}H_{89}N_2O_2S_4$ $[M+H]^+$ 853.5807, found 853.5812.

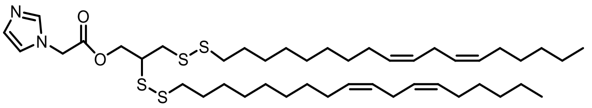

**2,3-bis(((9Z,12Z)-octadeca-9,12-dien-1-yl)disulfanyl)propyl 2-(1H-imidazol-1-yl)acetate (C2S18A)**

Synthesized by using the general procedure. $^1$H NMR (500 MHz, $CDCl_3$) $\delta$ = 7.51 (s, 1H), 7.10 (s, 1H), 6.96 (s, 1H), 5.42 – 5.29 (m, 8H), 4.74 (s, 2H), 4.49 (qd, *J* = 11.6, 5.6 Hz, 2H), 3.29 (dq, *J* = 8.6, 5.7 Hz, 1H), 3.02 (dd, *J* = 13.8, 5.8 Hz, 1H), 2.83 – 2.75 (m, 5H), 2.70 (td, *J* = 7.5, 2.6 Hz, 4H), 2.05 (q, *J* = 6.9 Hz, 8H), 1.72 – 1.62 (m, 4H), 1.40 – 1.25 (m, 32H), 0.89 (t, *J* = 6.9 Hz, 6H). $^{13}$C NMR (101 MHz, $CDCl_3$) $\delta$ = 167.18, 138.07, 130.36, 130.18, 129.97, 128.17, 128.02, 120.11, 65.50, 48.74, 48.09, 40.00, 39.78, 38.92, 31.67, 29.78, 29.56, 29.55, 29.49, 29.38, 29.35, 29.34, 29.29, 28.62, 28.59, 27.34, 25.77, 22.72, 14.24. **HR-MS** (ESI) *m/z* calcd for $C_{44}H_{77}O_2N_2S_4$ $[M+H]^+$ 793.4862, found 793.48682.

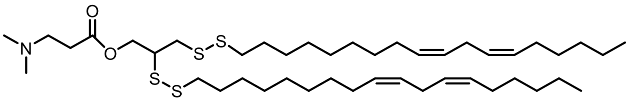

**2,3-bis(((9Z,12Z)-octadeca-9,12-dien-1-yl)disulfanyl)propyl 3-(dimethylamino) propanoate (C3S18A)**

Synthesized by using the general procedure. $^1$H NMR (400 MHz, $CDCl_3$) $\delta$ = 5.47 – 5.26 (m, 8H), 4.45 – 4.32 (m, 2H), 3.33 – 3.24 (m, 1H), 3.04 (dd, *J* = 13.2, 6.8 Hz, 1H), 2.94 (dd, *J* = 13.6, 8.0 Hz, 1H), 2.77 (t, *J* = 6.3 Hz, 4H), 2.70 (t, *J* = 7.3 Hz, 4H), 2.62 (t, *J* = 7.0 Hz, 2H), 2.51 (t, *J* = 7.2 Hz, 2H), 2.24 (s, 3H), 2.04 (dd, *J* = 13.5, 6.7 Hz, 8H), 1.73 – 1.61 (m, 8H), 1.41 – 1.24 (m, 32H), 0.89 (t, *J* = 6.2 Hz, 6H). $^{13}$C NMR (101 MHz, $CDCl_3$) $\delta$ = 172.22, 130.36, 130.22, 128.17, 128.05, 64.32, 54.83, 49.29, 45.41, 40.21, 39.97, 39.01, 32.98, 31.68, 29.81, 29.58, 29.50, 29.40, 29.37, 29.35, 29.31, 28.67, 28.63, 27.41, 27.37, 27.36, 25.78, 22.74, 14.25. **HR-MS** (ESI) *m/z* calcd for $C_{44}H_{82}O_2NS_4$ $[M+H]^+$ 784.5223, found 784.5222.

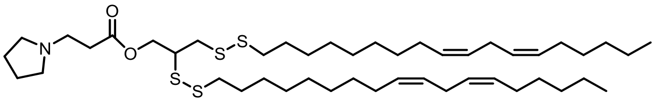

**2,3-bis(((9Z,12Z)-octadeca-9,12-dien-1-yl)disulfanyl)propyl 3-(pyrrolidin-1-yl)propanoate (C3AS18A)**

Synthesized by using the general procedure. $^1$H NMR (400 MHz, $CDCl_3$) $\delta$ = 5.42 – 5.29 (m, 8H), 4.38 (qd, *J* = 11.6, 5.6 Hz, 2H), 3.28 (dq, *J* = 11.9, 5.8 Hz, 1H), 3.04 (dd, *J* = 13.8, 6.3 Hz, 1H), 2.94 (dd, *J* = 13.8, 8.0 Hz, 1H), 2.83 – 2.75 (m, 6H), 2.70 (t, *J* = 7.3 Hz, 4H), 2.57 (t, *J* = 7.5 Hz, 2H), 2.53 (dd, *J* = 9.2, 3.9 Hz, 4H), 2.05 (q, *J* = 6.8 Hz, 8H), 1.81 – 1.75 (m, 4H), 1.72 – 1.62 (m, 4H), 1.44 – 1.22 (m, 32H), 0.89 (t, *J* = 6.9 Hz, 6H). $^{13}$C NMR (101 MHz, $CDCl_3$) $\delta$ = 172.22, 130.35, 130.21, 128.16, 128.04, 64.24, 54.14, 51.46, 49.32, 40.16, 39.96, 39.00, 34.23, 31.68, 29.80, 29.57, 29.50, 29.40, 29.37, 29.34, 29.31, 28.66, 28.63, 27.37, 27.35, 25.78, 23.63, 22.73, 14.24. **HR-MS** (ESI) *m/z* calcd for $C_{46}H_{84}O_2NS_4$ $[M+H]^+$ 810.5379, found 810.5373.

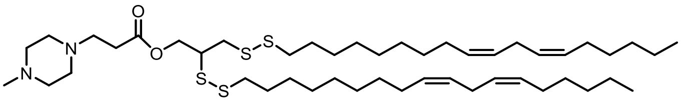

**2,3-bis(((9Z,12Z)-octadeca-9,12-dien-1-yl)disulfanyl)propyl3-(4-methylpiperazin-1-yl)propanoate (C3BS18A)**

Synthesized by using the general procedure. $^1$H NMR (500 MHz, $CDCl_3$) $\delta$ = 5.41 – 5.30 (m, 8H), 4.41 (dd, *J* = 11.6, 5.7 Hz, 1H), 4.35 (dd, *J* = 11.6, 5.4 Hz, 1H), 3.28 (dq, *J* = 7.9,

5.7 Hz, 1H), 3.04 (dd, *J* = 13.7, 6.3 Hz, 1H), 2.94 (dd, *J* = 13.8, 8.1 Hz, 1H), 2.77 (t, *J* = 6.7 Hz, 4H), 2.73 – 2.65 (m, 7H), 2.57 – 2.41 (m, 6H), 2.28 (s, 3H), 2.08 – 2.01 (m, 8H), 1.80 – 1.71 (m, 3H), 1.66 (dt, *J* = 14.6, 7.3 Hz, 4H), 1.41 – 1.24 (m, 32H), 0.89 (t, *J* = 6.9 Hz, 6H). $^{13}$C NMR (101 MHz, $CDCl_3$) $\delta$ = 172.19, 130.36, 130.20, 128.16, 128.03, 64.18, 55.13, 53.58, 52.94, 49.29, 46.10, 40.11, 39.95, 38.98, 32.43, 31.67, 29.80, 29.57, 29.49, 29.40, 29.37, 29.36, 29.33, 29.31, 28.66, 28.63, 27.36, 27.35, 25.77, 22.73, 14.24. **HR-MS** (ESI) *m/z* calcd for $C_{47}H_{87}O_2N_2S_4$ $[M+H]^+$ 839.5645, found 839.5651.

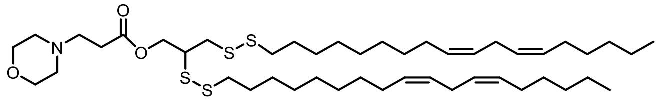

**2,3-bis(((9Z,12Z)-octadeca-9,12-dien-1-yl)disulfanyl)propyl 3-morpholino-propanoate (C3CS18A)**

Synthesized by using the general procedure. $^{1}$H NMR (400 MHz, $CDCl_3$) $\delta$ = 5.44 – 5.27 (m, 8H), 4.39 (qd, *J* = 11.6, 5.5 Hz, 2H), 3.71 – 3.68 (m, 4H), 3.28 (dq, *J* = 8.1, 5.7 Hz, 1H), 3.05 (dd, *J* = 13.7, 6.1 Hz, 1H), 2.93 (dd, *J* = 13.7, 8.2 Hz, 1H), 2.77 (t, *J* = 6.5 Hz, 4H), 2.70 (dd, *J* = 14.8, 7.3 Hz, 6H), 2.53 (t, *J* = 7.1 Hz, 2H), 2.49 –2.46 (m, 2H), 2.05 (dd, *J* = 13.7, 6.8 Hz, 8H), 1.67 (ddd, *J* = 12.9, 10.6, 5.6 Hz, 6H), 1.32 (td, *J* = 13.3, 8.5 Hz, 32H), 0.89 (t, *J* = 6.8 Hz, 6H). $^{13}$C NMR (101 MHz, $CDCl_3$) $\delta$ = 171.96, 130.23, 130.07, 128.04, 127.91, 66.93, 64.05, 53.92, 53.40, 49.16, 39.99, 39.84, 38.88, 32.12, 31.55, 29.67, 29.44, 29.37, 29.27, 29.23, 29.21, 29.19, 28.53, 28.50, 27.22, 25.65, 22.60, 14.11. **HR-MS** (ESI) *m/z* calcd for $C_{46}H_{84}O_3NS_4$ $[M+H]^+$ 826.5329, found 826.5330.

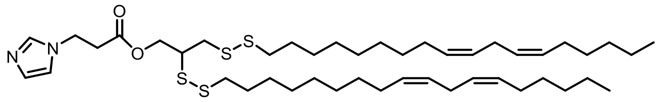

**2,3-bis(((9Z,12Z)-octadeca-9,12-dien-1-yl)disulfanyl)propyl 3-(1H-imidazol-1-yl)propanoate (C3DS18A)**

Synthesized by using the general procedure. $^{1}$H NMR (400 MHz, $CDCl_3$) $\delta$ = 7.52 (s, 1H), 7.05 (s, 1H), 6.94 (s, 1H), 5.43 – 5.28 (m, 8H), 4.40 (d, *J* = 5.6 Hz, 2H), 4.27 (t, *J* = 6.6 Hz, 2H), 3.26 (dq, *J* = 8.3, 5.7 Hz, 1H), 3.02 (dd, *J* = 13.8, 6.0 Hz, 1H), 2.85 – 2.75 (m, 6H), 2.69 (td, *J* = 7.5, 2.8 Hz, 3H), 2.09 – 1.99 (m, 8H), 1.65 (dt, *J* = 14.6, 7.3 Hz, 4H), 1.41 – 1.24 (m, 34H), 0.89 (t, *J* = 6.8 Hz, 6H).$^{13}$C NMR (101 MHz, $CDCl_3$) $\delta$ = 170.29, 137.39, 130.36, 130.19, 129.92, 128.18, 128.03, 118.93, 64.81, 48.97, 42.28, 40.00, 38.97, 36.00, 31.67, 29.79, 29.56, 29.49, 29.39, 29.35, 29.34, 29.29, 28.64, 28.60, 27.35, 25.77, 22.72, 14.24. **HR-MS** (ESI) *m/z* calcd for $C_{45}H_{79}O_2N_2S_4$ $[M+H]^+$ 807.5019, found 807.5018.

## References

[1] a) Jayaraman, M.; Ansell, S. M.; Mui, B. L.; Tam, Y. K.; Chen, J.; Du, X.; Butler, D.; Eltepu, L.; Matsuda, S.; Narayanannair, J. K.; Rajeev, K. G.; Hafez, I. M.; Akinc, A.; Maier, M. A.; Tracy, M. A.; Cullis, P. R.; Madden, T. D.; Manoharan, M.; Hope, M. J. Maximizing the potency of siRNA lipid nanoparticles for hepatic gene silencing in vivo. *Angew. Chem. Int. Ed.* **2012**, *51* (34), 8529; b) Sabnis, S.; Kumarasinghe, E. S.; Salerno, T.; Mihai, C.; Ketova, T.; Senn, J. J.; Lynn, A.; Bulychev, A.; McFadyen, I.; Chan, J.; Almarsson, O.; Stanton, M. G.; Benenato, K. E. A Novel amino lipid series for mRNA delivery: improved endosomal escape and sustained pharmacology and safety in non-human primates. *Mol. Ther.* **2018**, *26* (6), 1509.

**NMR Spectrum**

**C4S10**
(500 MHz, $CDCl_3$)

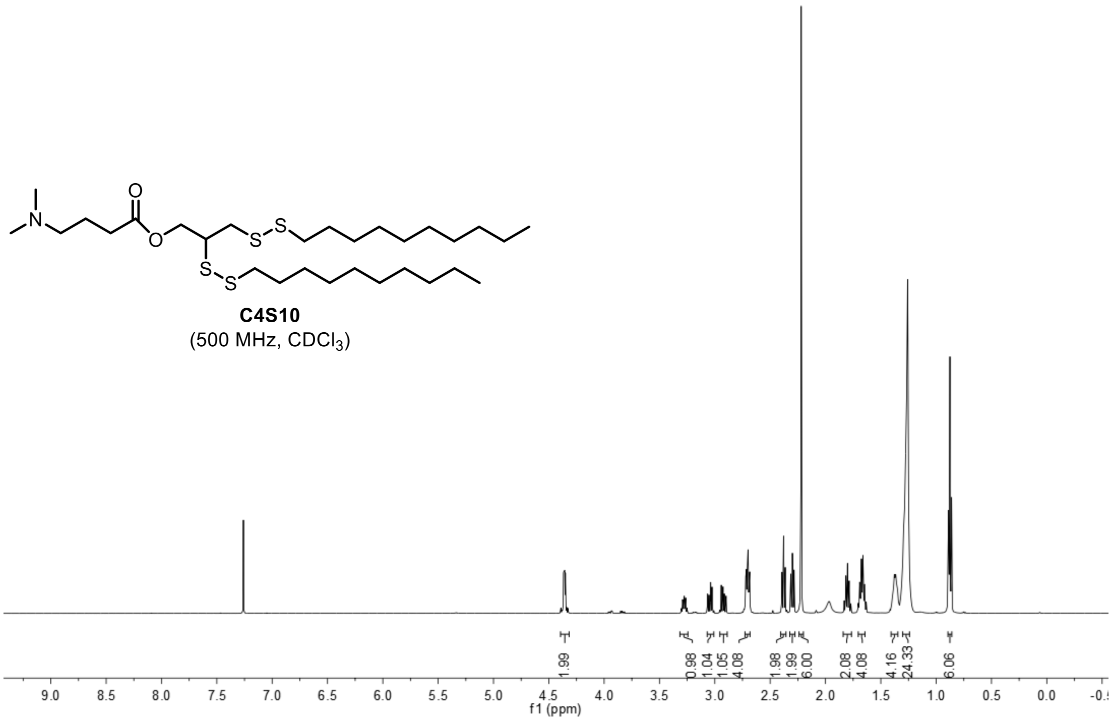


**C4S10**
(101 MHz, $CDCl_3$)

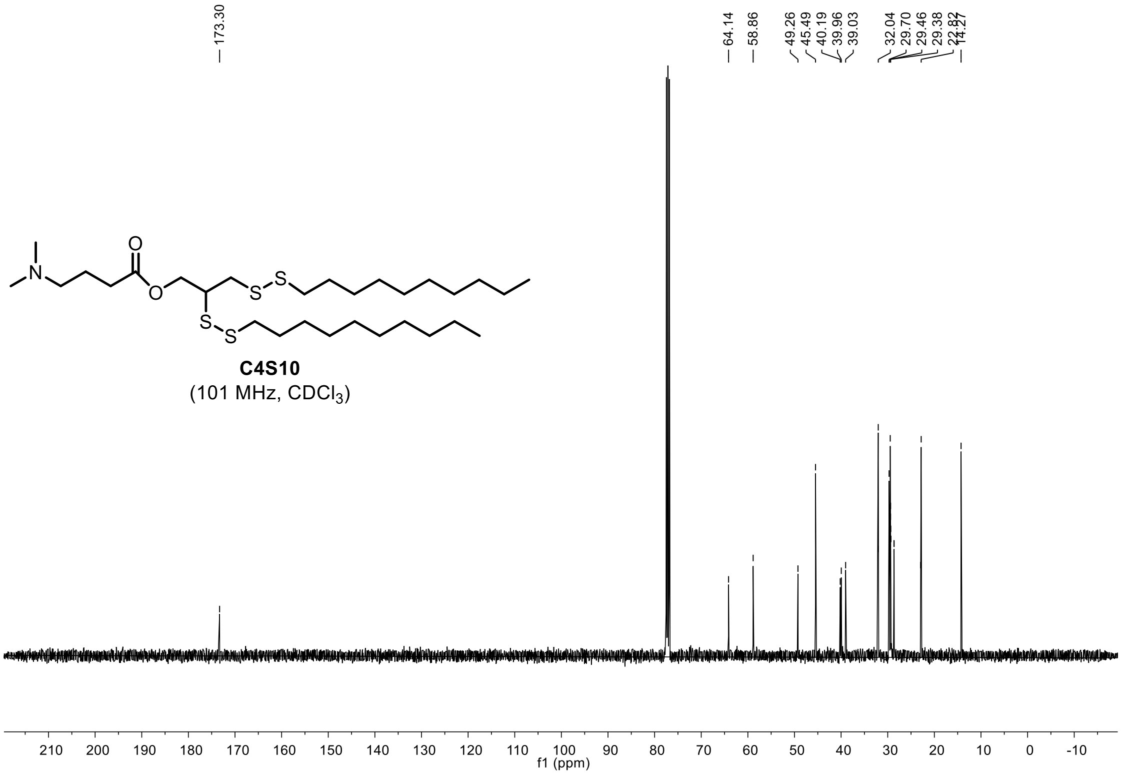

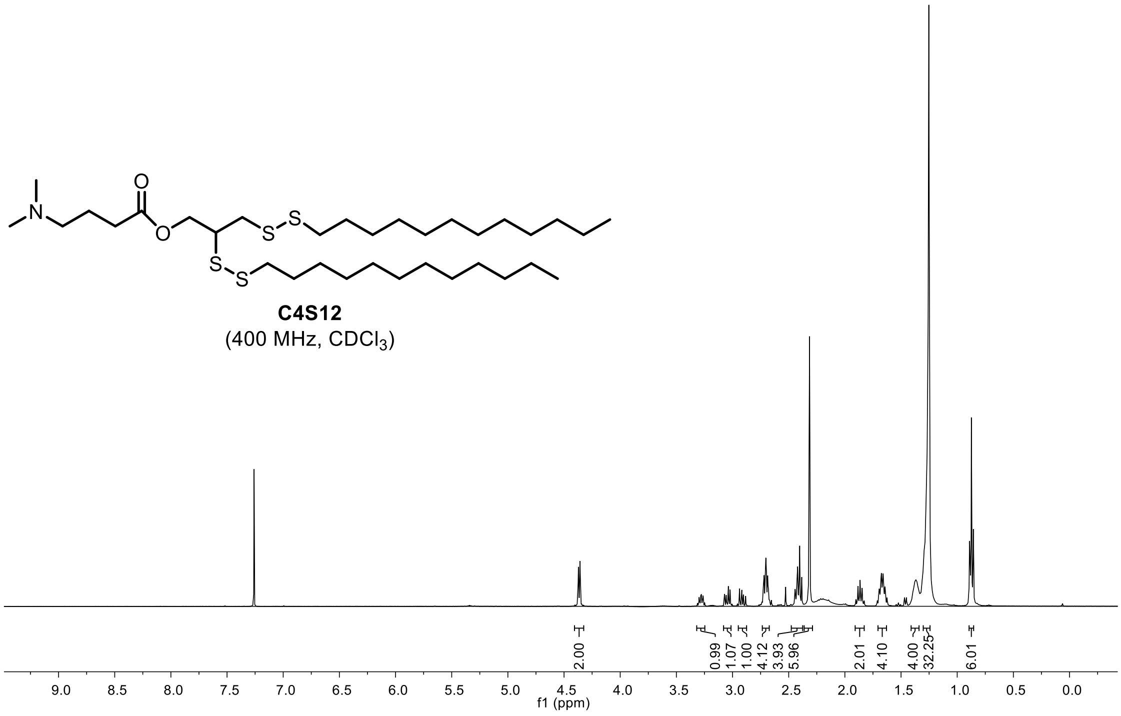
C4S12
(400 MHz, CDCl3)
2.00
0.99
1.07
1.00
4.12
3.93
5.96
2.01
4.10
4.00
32.25
6.01
f1 (ppm)

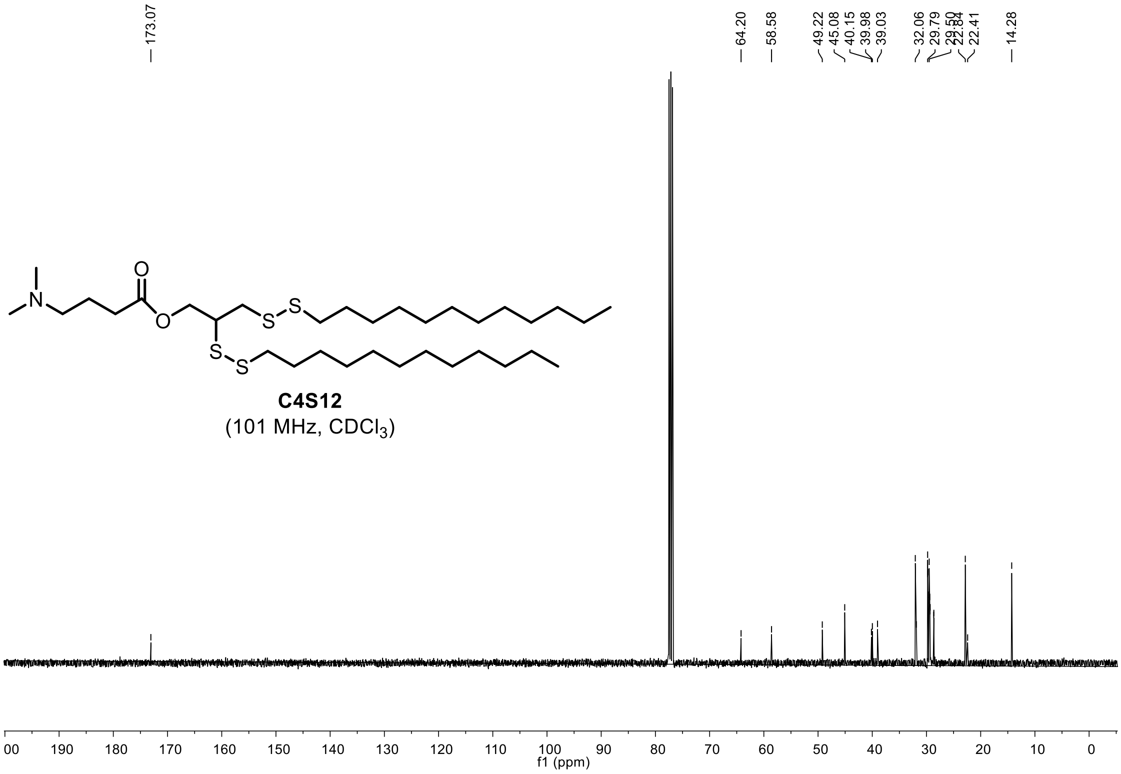
173.07
64.20
58.58
49.22
45.08
40.15
39.98
39.03
32.06
29.79
22.41
14.28
C4S12
(101 MHz, CDCl3)
f1 (ppm)

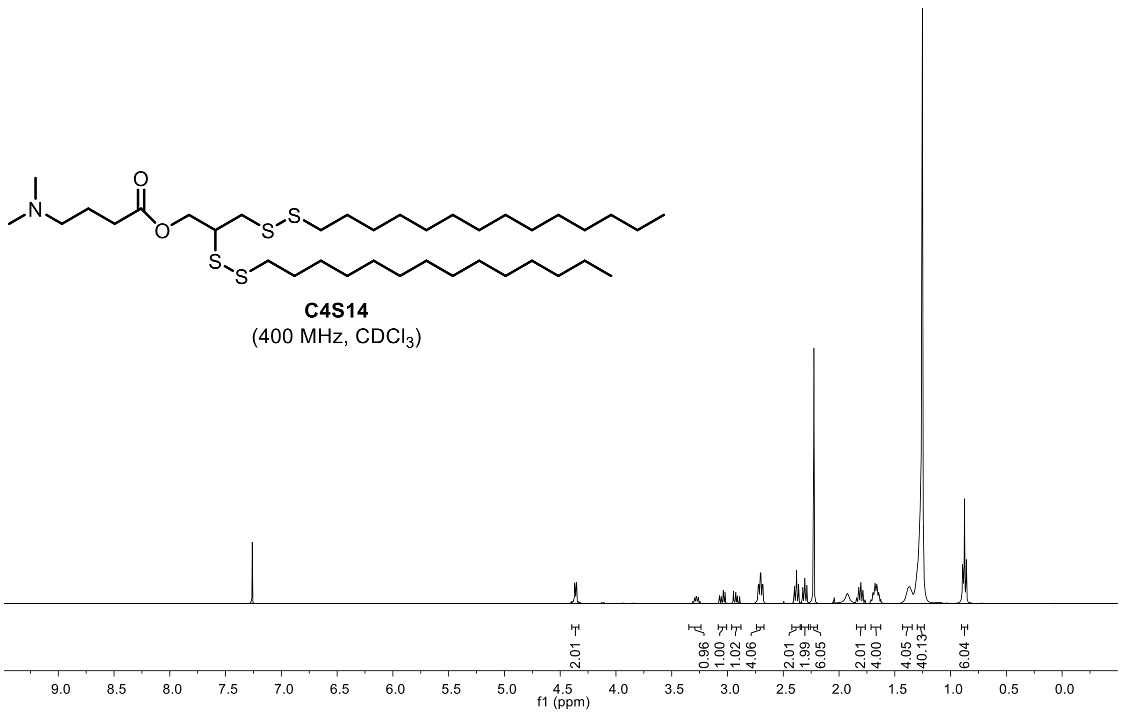

C4S14
(400 MHz, CDCl3)
2.01
0.96
1.00
1.02
4.06
2.01
1.99
6.05
2.01
4.00
4.05
40.13
6.04
f1 (ppm)


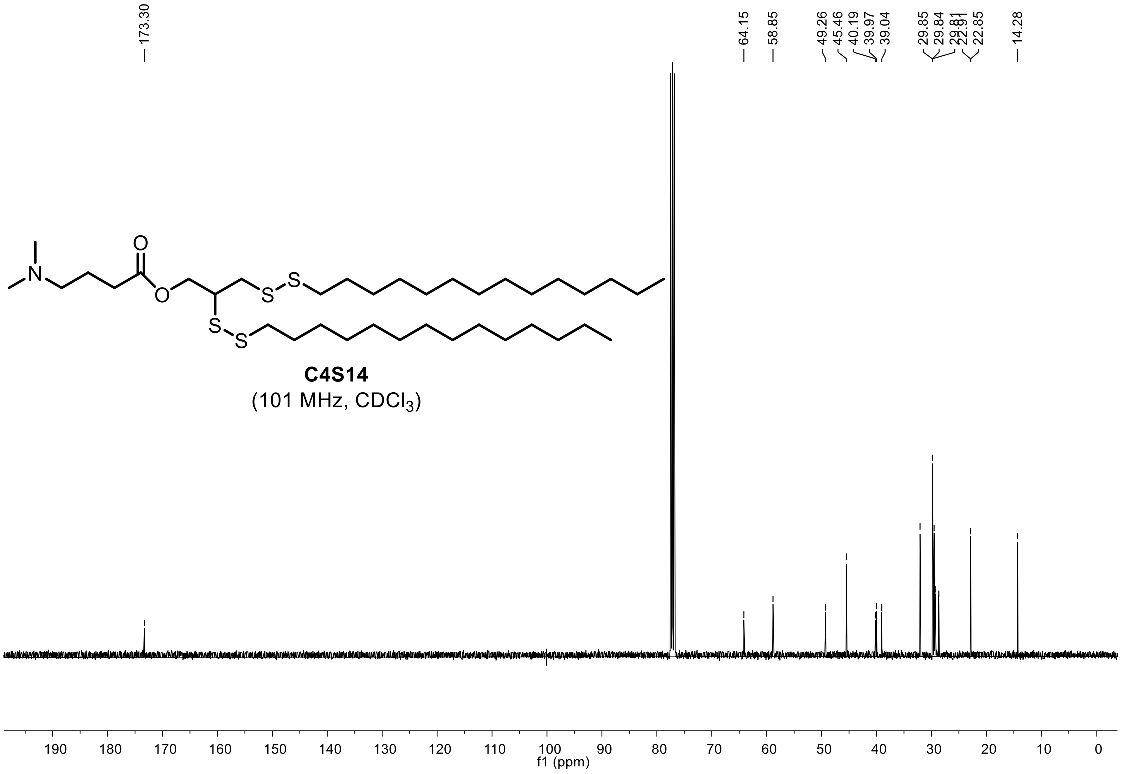

173.30
64.15
58.85
49.26
45.46
40.19
39.97
39.04
29.85
29.84
22.85
14.28
C4S14
(101 MHz, CDCl3)
f1 (ppm)

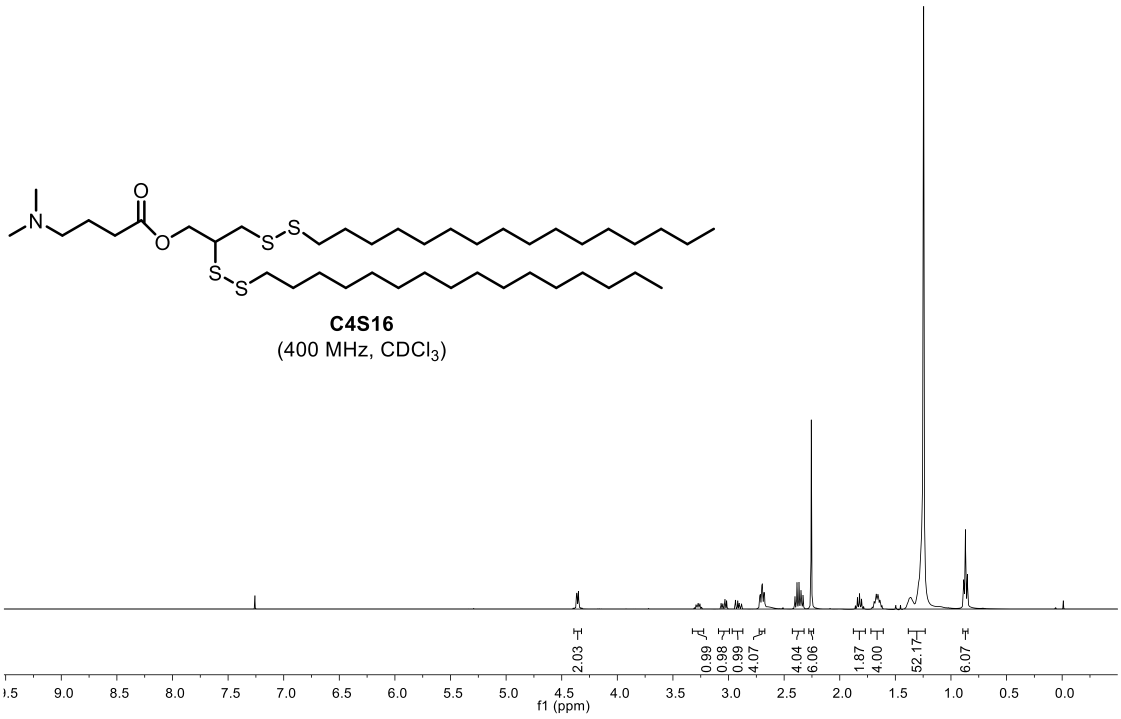

C4S16
(400 MHz, CDCl3)
2.03
0.99
0.98
0.99
4.07
4.04
6.06
1.87
4.00
52.17
6.07
f1 (ppm)


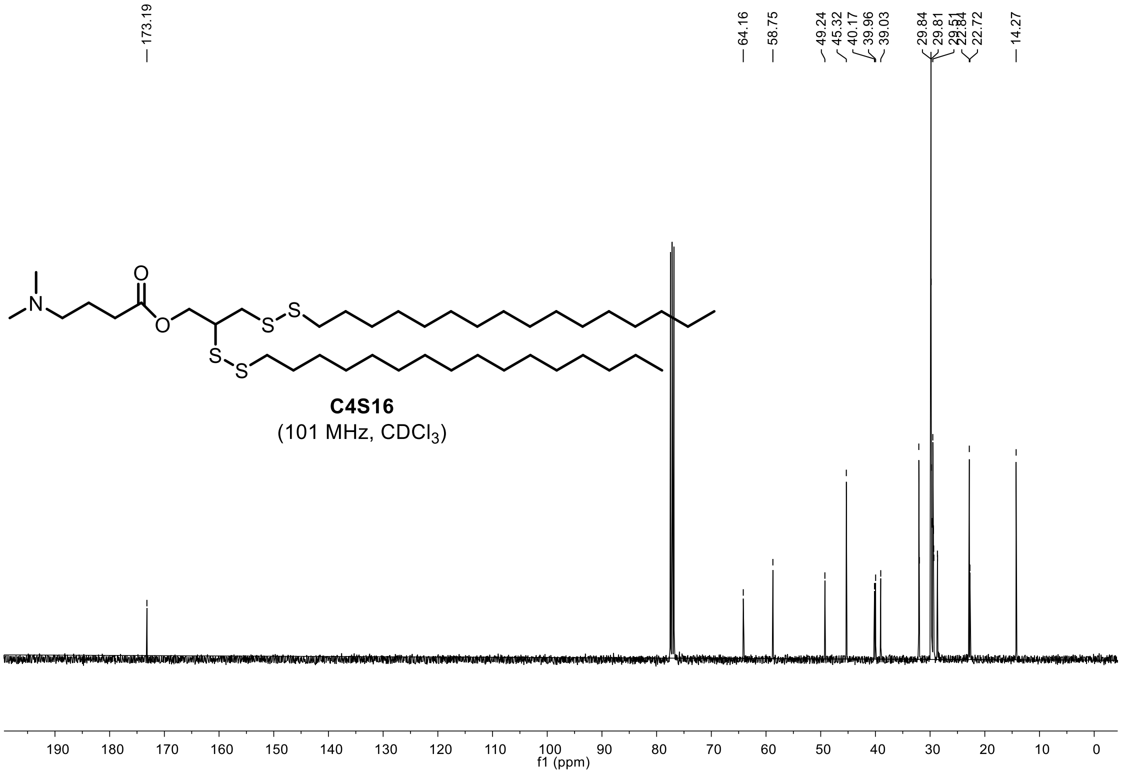

173.19
64.16
58.75
49.24
45.32
40.17
39.96
39.03
29.84
29.81
22.72
14.27
C4S16
(101 MHz, CDCl3)
f1 (ppm)

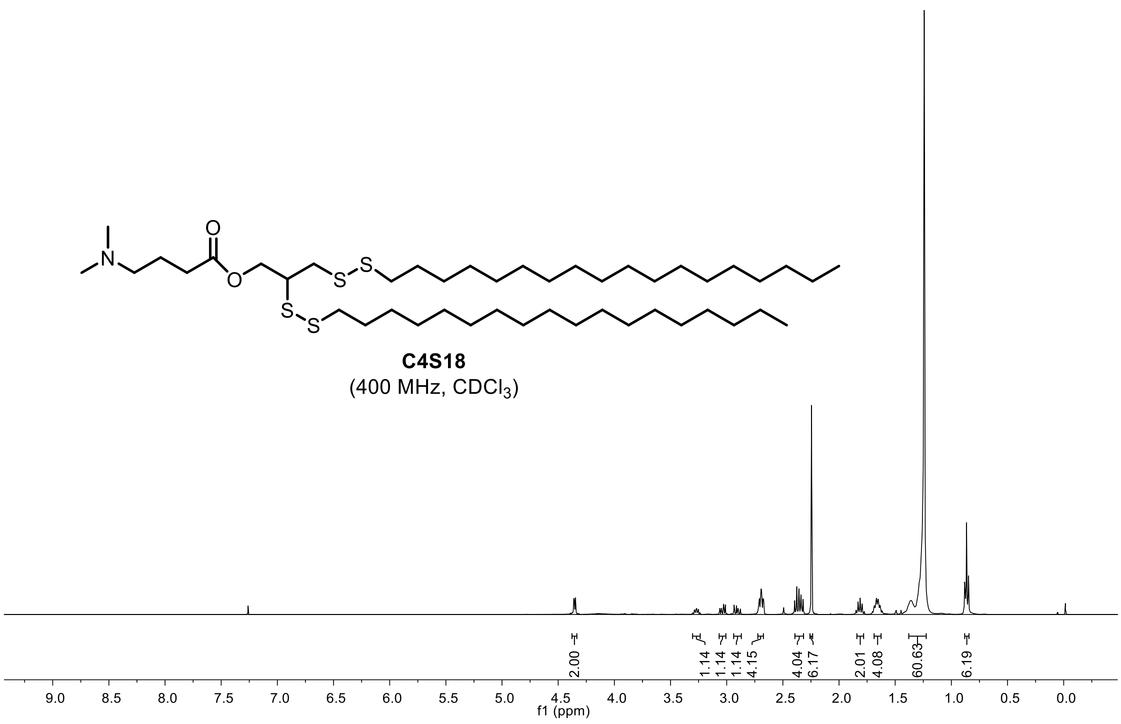
C4S18
(400 MHz, CDCl3)
2.00
1.14
1.14
1.14
4.15
4.04
6.17
2.01
4.08
60.63
6.19
9.0 8.5 8.0 7.5 7.0 6.5 6.0 5.5 5.0 4.5 4.0 3.5 3.0 2.5 2.0 1.5 1.0 0.5 0.0
f1 (ppm)

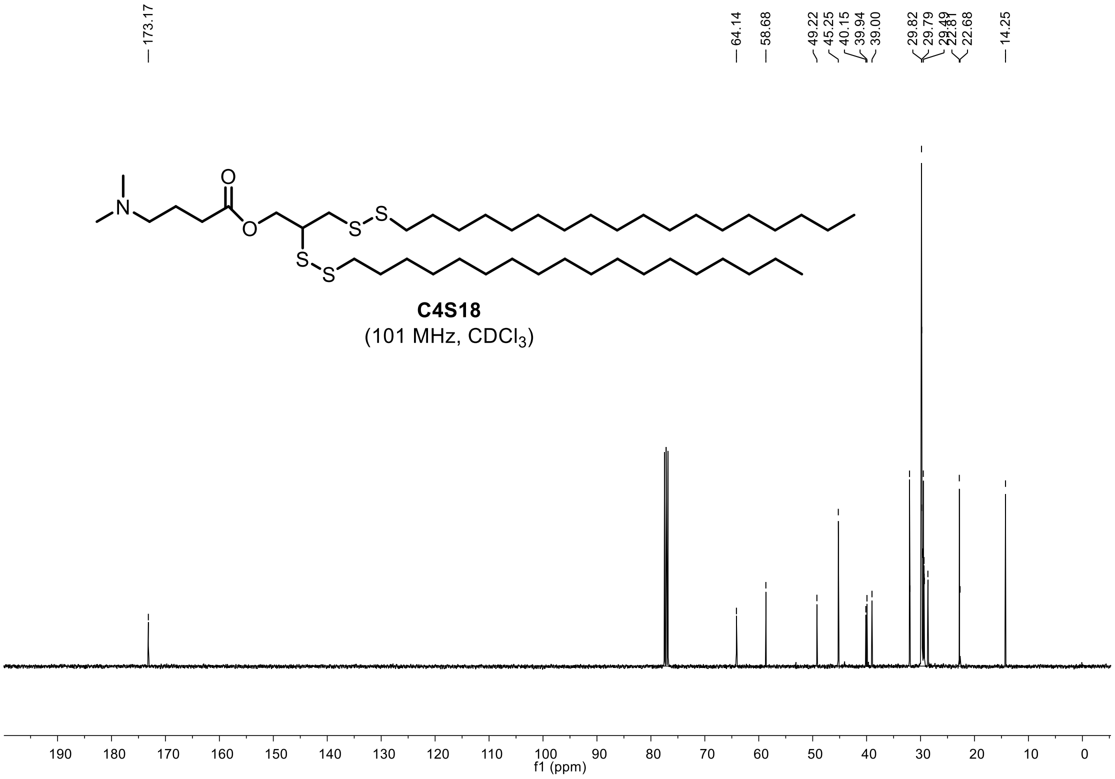
173.17
64.14
58.68
49.22
45.25
40.15
39.94
39.00
29.82
29.79
29.49
22.81
22.68
14.25
C4S18
(101 MHz, CDCl3)
190 180 170 160 150 140 130 120 110 100 90 80 70 60 50 40 30 20 10 0
f1 (ppm)

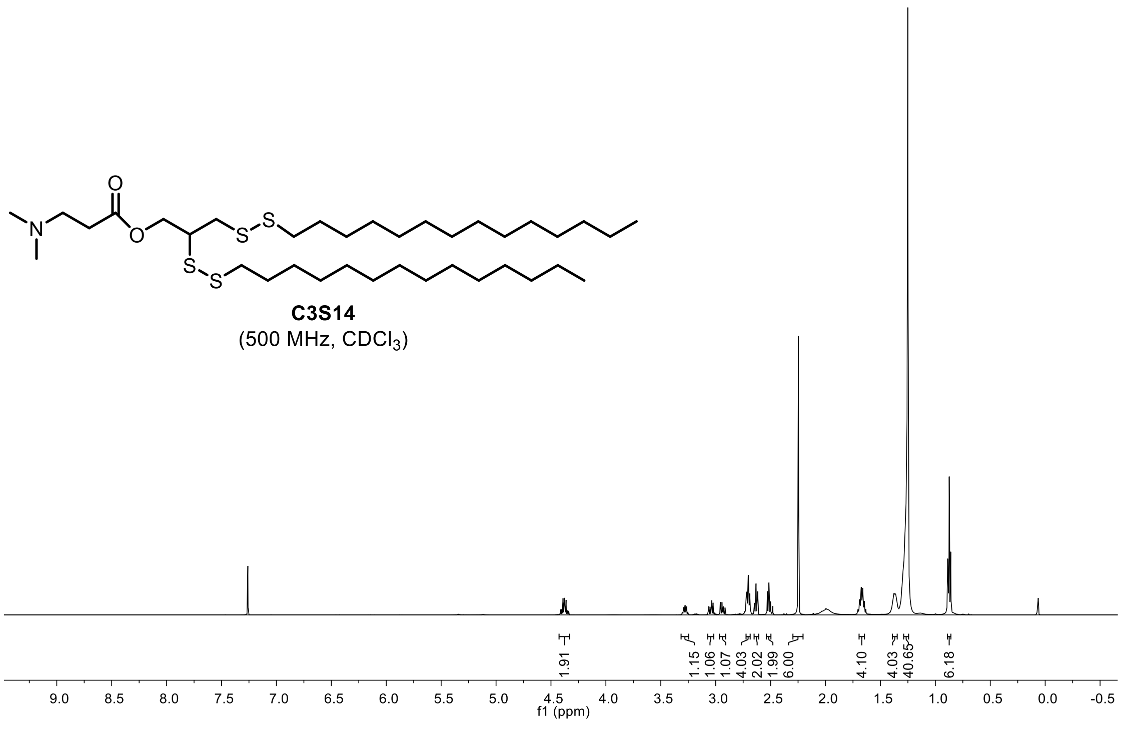
C3S14
(500 MHz, CDCl3)
1.91
1.15
1.06
1.07
4.03
2.02
1.99
6.00
4.10
4.03
40.65
6.18
f1 (ppm)

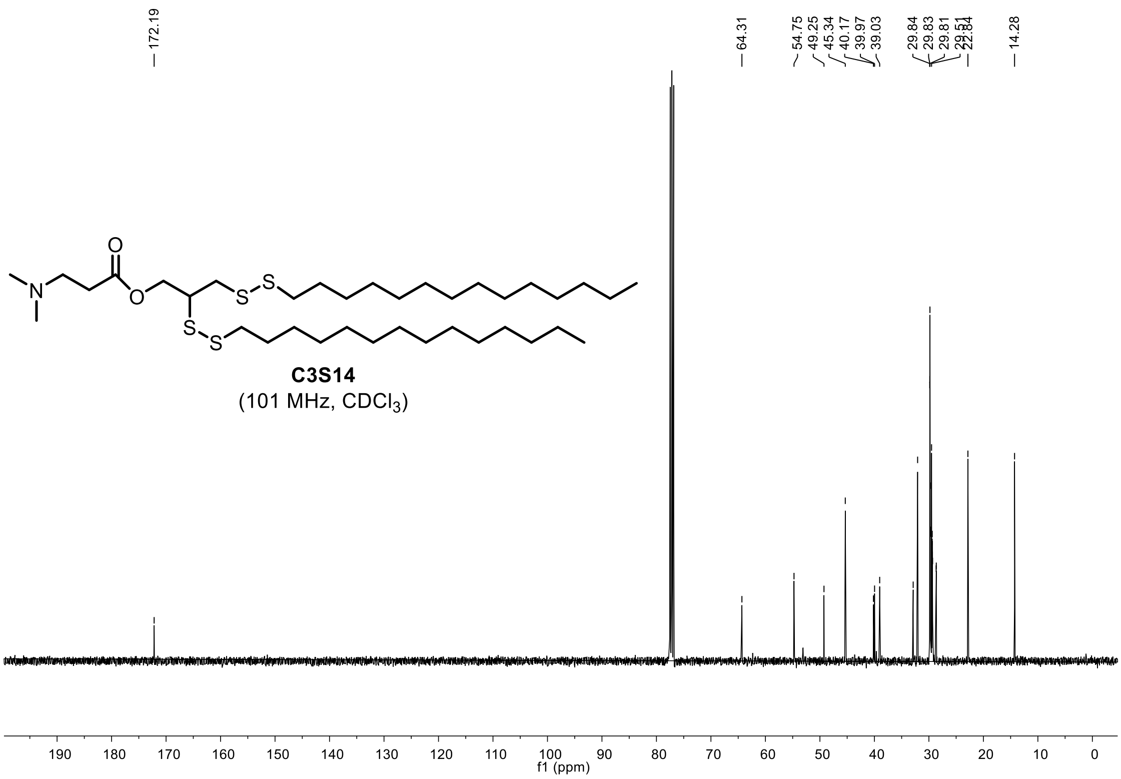
172.19
64.31
54.75
49.25
45.34
40.17
39.97
39.03
29.84
29.83
29.81
29.51
22.84
14.28
C3S14
(101 MHz, CDCl3)
f1 (ppm)

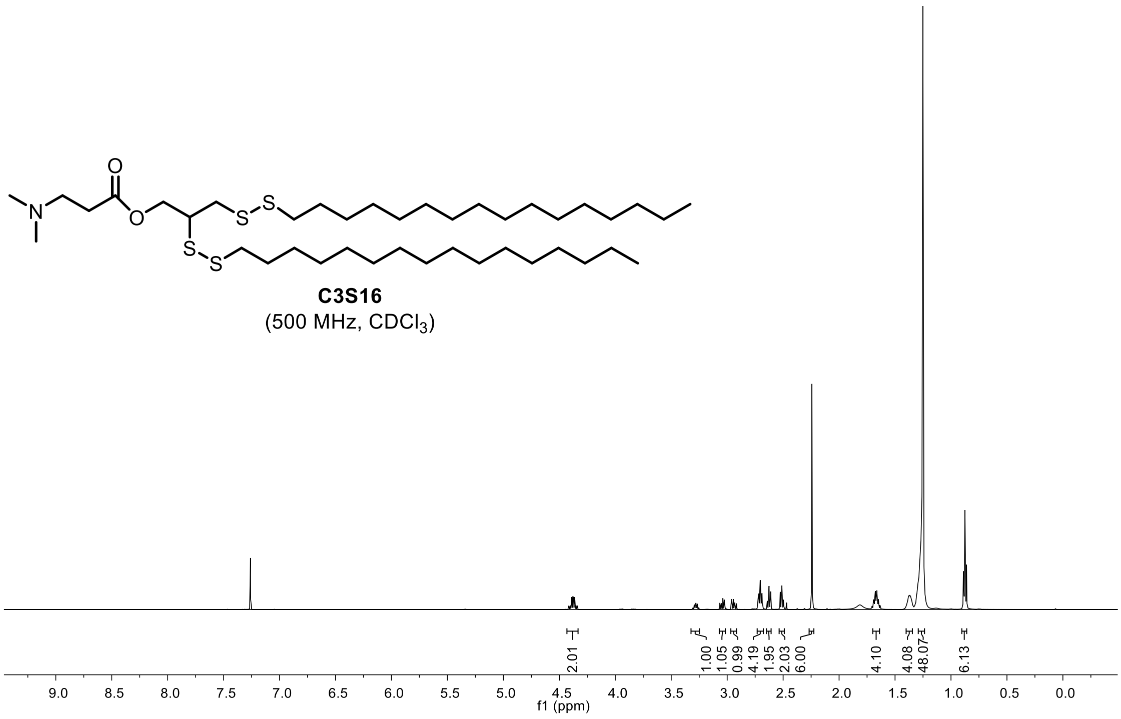

C3S16
(500 MHz, CDCl3)
2.01
1.00
1.05
0.99
4.19
1.95
2.03
6.00
4.10
4.08
48.07
6.13
9.0 8.5 8.0 7.5 7.0 6.5 6.0 5.5 5.0 4.5 4.0 3.5 3.0 2.5 2.0 1.5 1.0 0.5 0.0
f1 (ppm)


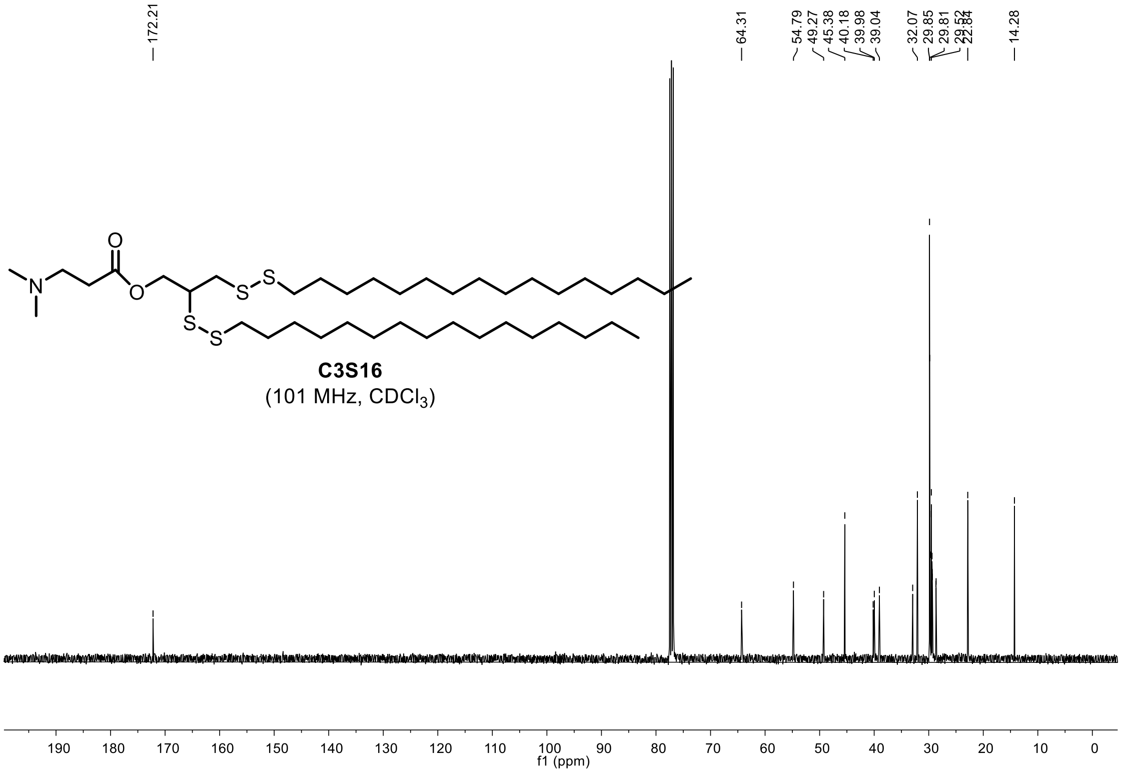

172.21
64.31
54.79
49.27
45.38
40.18
39.98
39.04
32.07
29.85
29.81
29.52
22.84
14.28
C3S16
(101 MHz, CDCl3)
190 180 170 160 150 140 130 120 110 100 90 80 70 60 50 40 30 20 10 0
f1 (ppm)

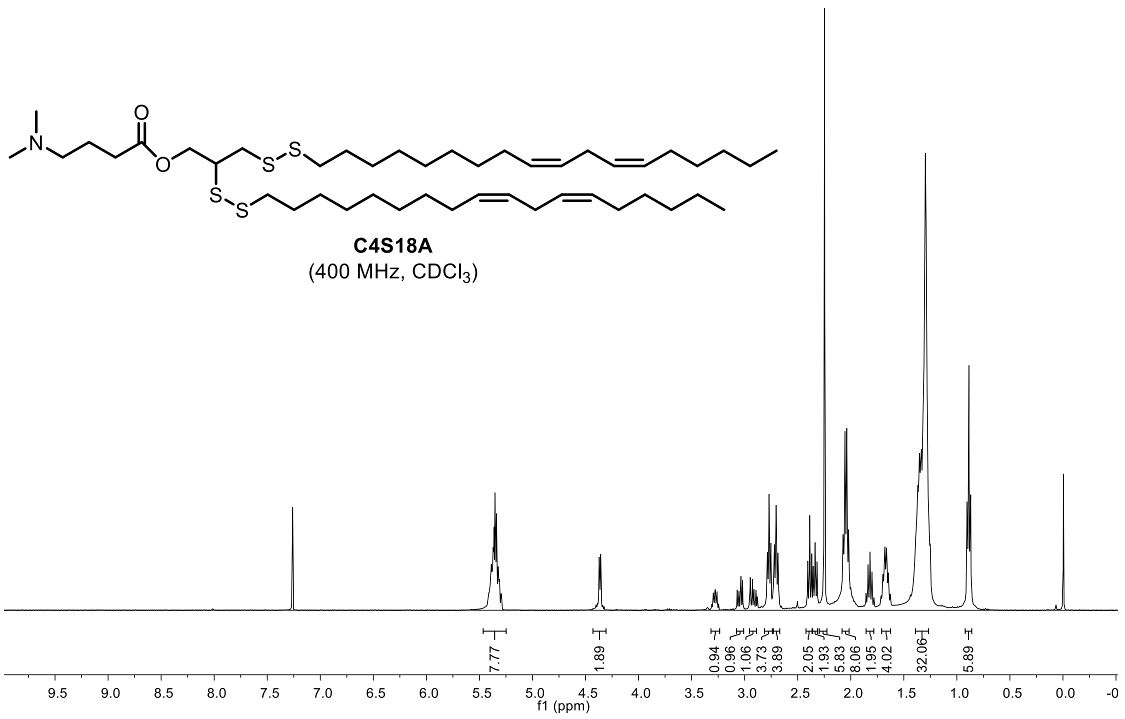
C4S18A
(400 MHz, CDCl$_3$)
7.77
1.89
0.94
0.96
1.06
3.73
3.89
2.05
1.93
5.83
8.06
1.95
4.02
32.06
5.89
9.5 9.0 8.5 8.0 7.5 7.0 6.5 6.0 5.5 5.0 4.5 4.0 3.5 3.0 2.5 2.0 1.5 1.0 0.5 0.0 -0
f1 (ppm)

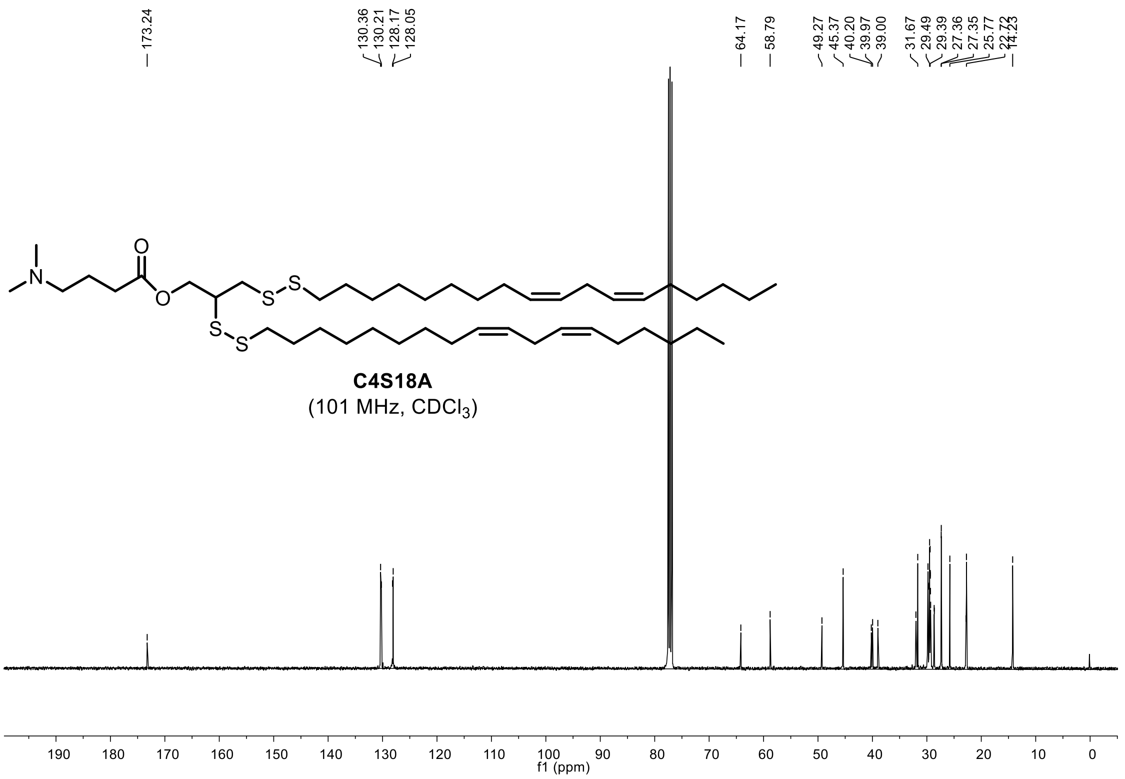
173.24
130.36
130.21
128.17
128.05
64.17
58.79
49.27
45.37
40.20
39.97
39.00
31.67
29.49
29.39
27.36
27.35
25.77
22.72
14.23
C4S18A
(101 MHz, CDCl$_3$)
190 180 170 160 150 140 130 120 110 100 90 80 70 60 50 40 30 20 10 0
f1 (ppm)

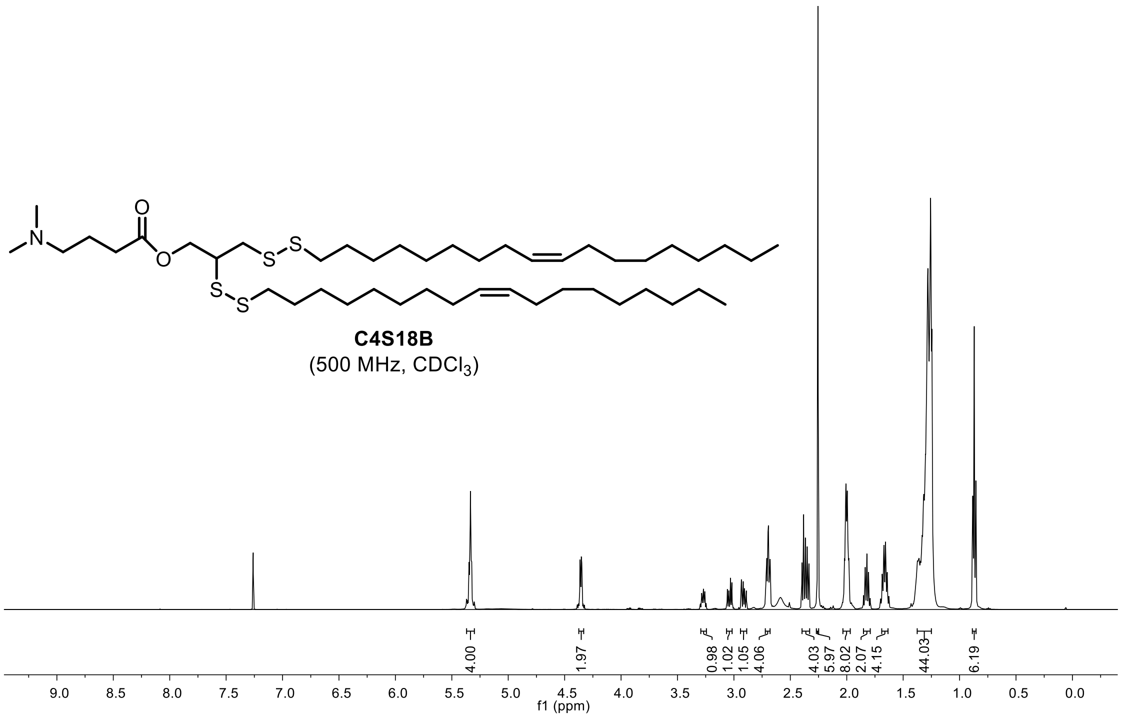
C4S18B
(500 MHz, CDCl3)
4.00
1.97
0.98
1.02
1.05
4.06
4.03
5.97
8.02
2.07
4.15
44.03
6.19
f1 (ppm)

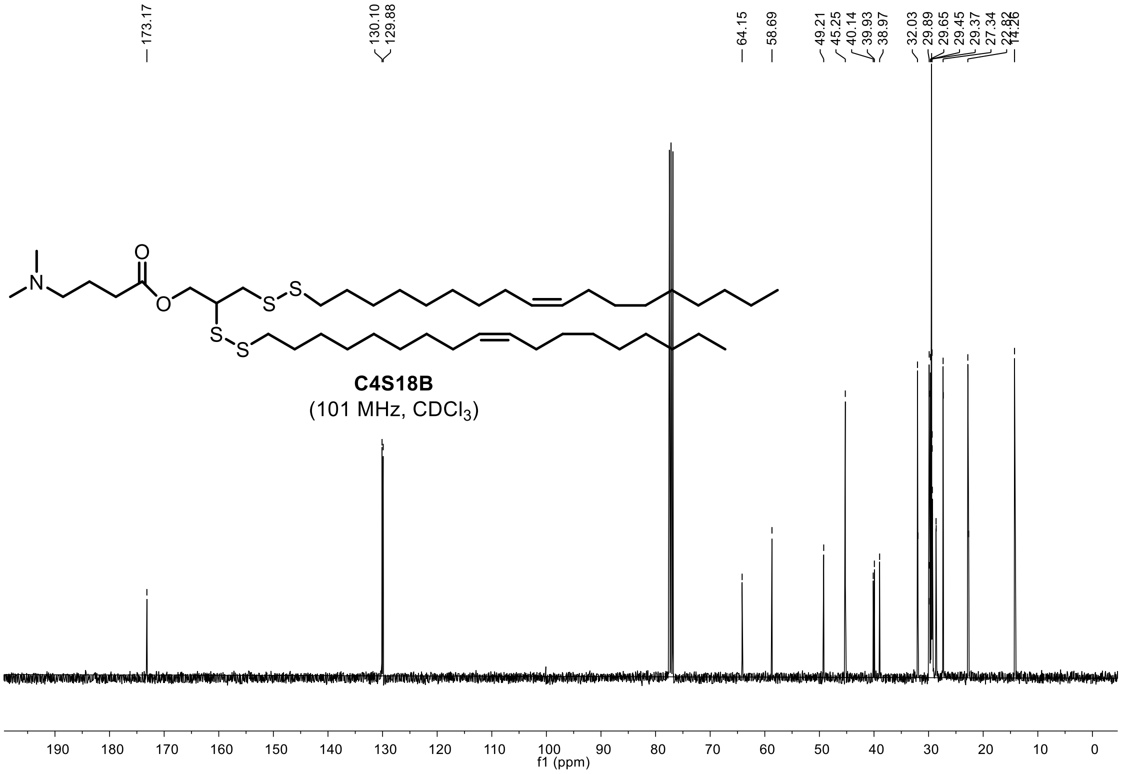
173.17
130.10
129.88
64.15
58.69
49.21
45.25
40.14
39.93
38.97
32.03
29.89
29.65
29.45
29.37
27.34
C4S18B
(101 MHz, CDCl3)
f1 (ppm)

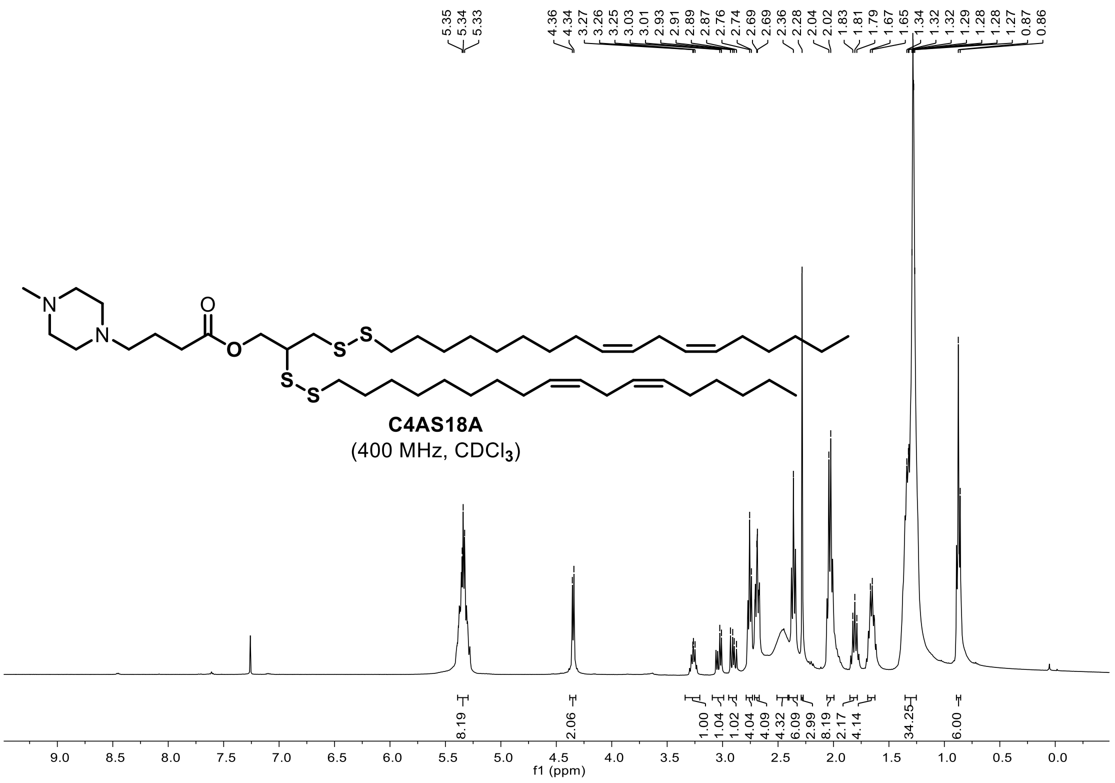
5.35
5.34
5.33
4.36
4.34
3.27
3.26
3.25
3.03
3.01
2.93
2.91
2.89
2.87
2.76
2.74
2.69
2.69
2.36
2.28
2.04
2.02
1.83
1.81
1.79
1.67
1.65
1.34
1.32
1.29
1.28
1.28
1.27
0.87
0.86
C4AS18A
(400 MHz, CDCl3)
8.19
2.06
1.00
1.04
1.02
4.04
4.09
4.32
6.09
2.99
8.19
2.17
4.14
34.25
6.00
f1 (ppm)

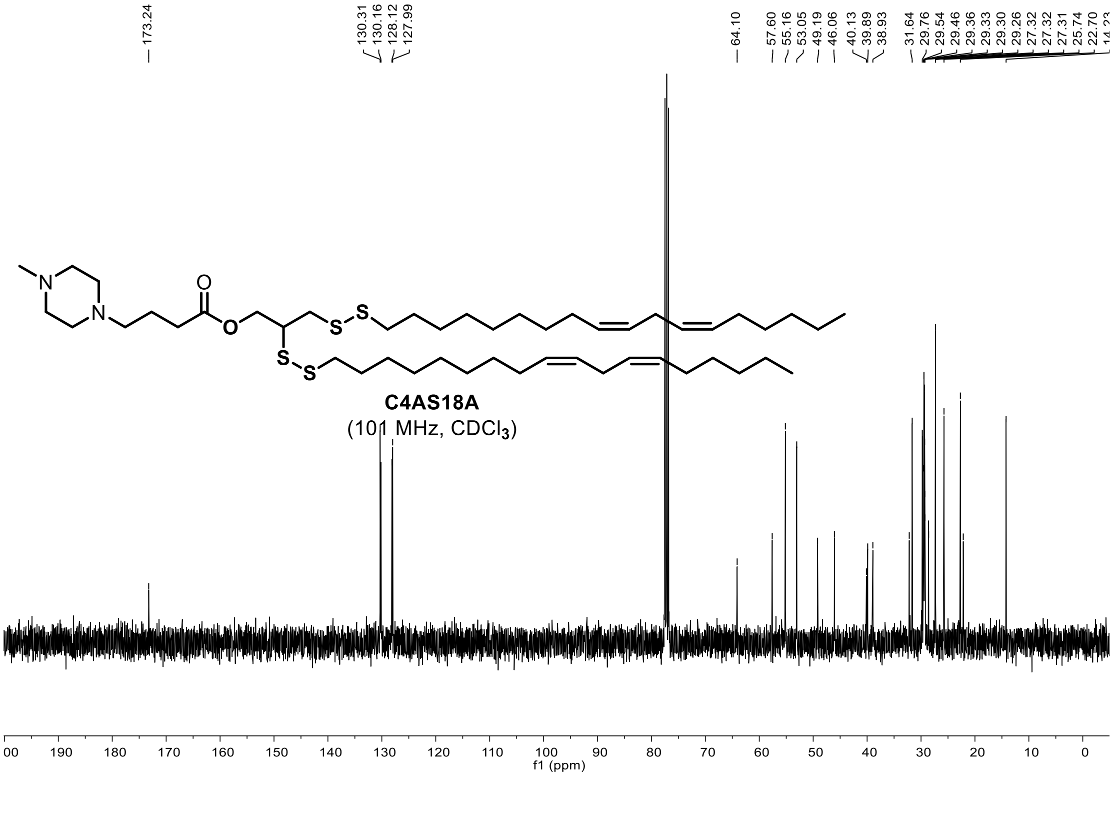
173.24
130.31
130.16
128.12
127.99
64.10
57.60
55.16
53.05
49.19
46.06
40.13
39.89
38.93
31.64
29.76
29.54
29.46
29.36
29.33
29.30
29.26
27.32
27.31
25.74
22.70
C4AS18A
(101 MHz, CDCl3)
f1 (ppm)

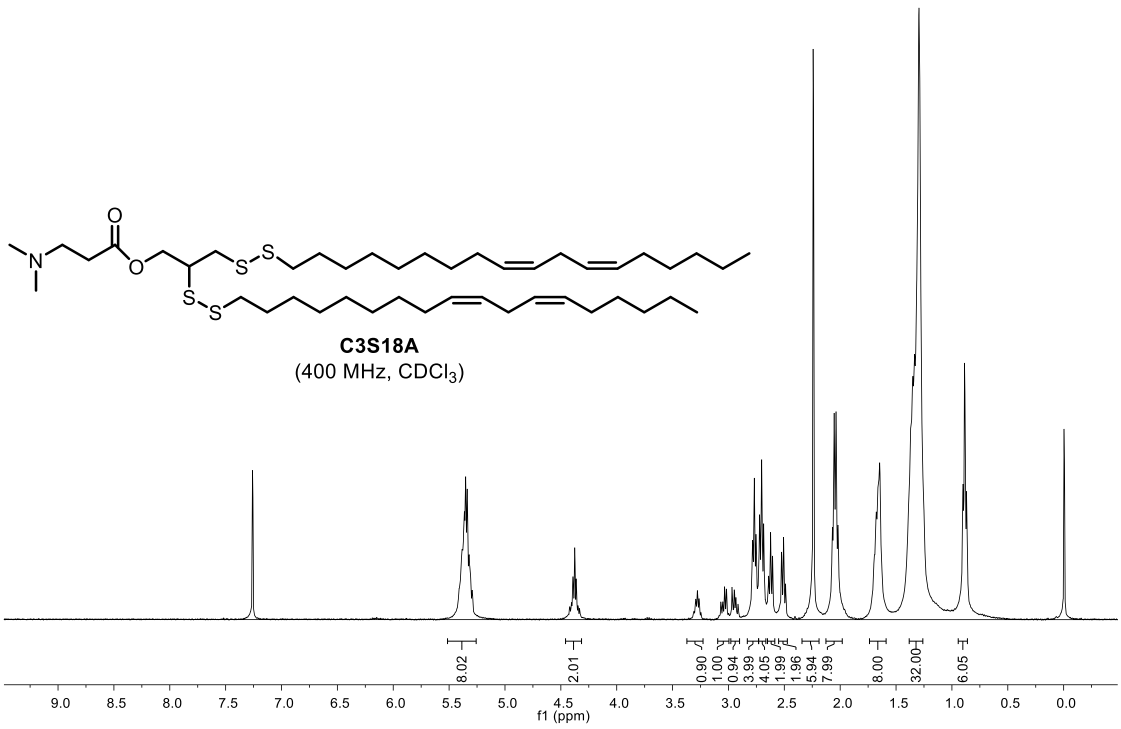
C3S18A
(400 MHz, CDCl3)
8.02
2.01
0.90
1.00
0.94
3.99
4.05
1.99
1.96
5.94
7.99
8.00
32.00
6.05
f1 (ppm)

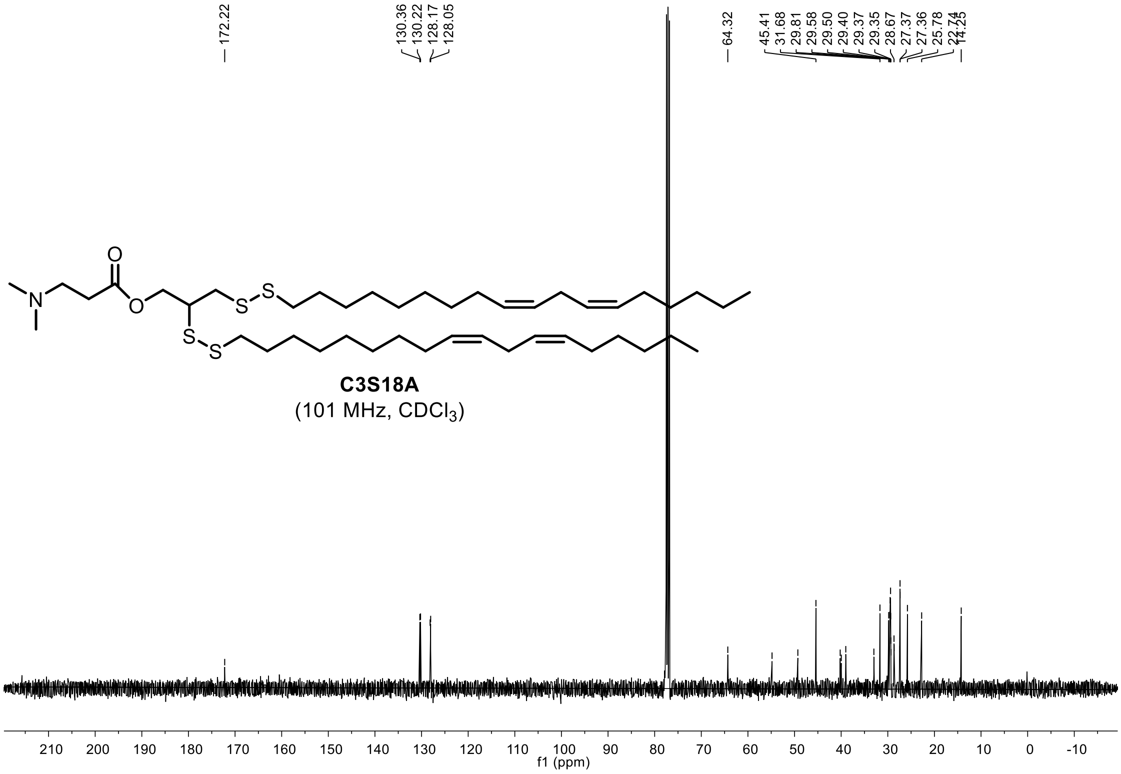
172.22
130.36
130.22
128.17
128.05
64.32
45.41
31.68
29.81
29.58
29.50
29.40
29.37
29.35
28.67
27.37
27.36
25.78
14.25
C3S18A
(101 MHz, CDCl3)
f1 (ppm)

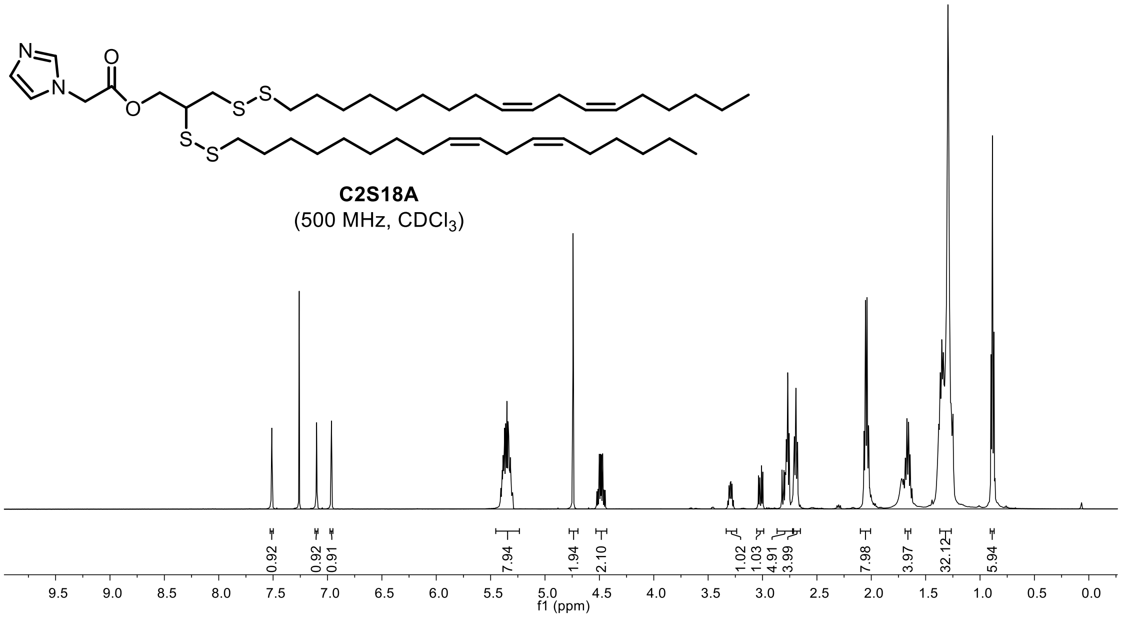

C2S18A
(500 MHz, $CDCl_3$)
0.92
0.92
0.91
7.94
1.94
2.10
1.02
1.03
4.91
3.99
7.98
3.97
32.12
5.94
f1 (ppm)


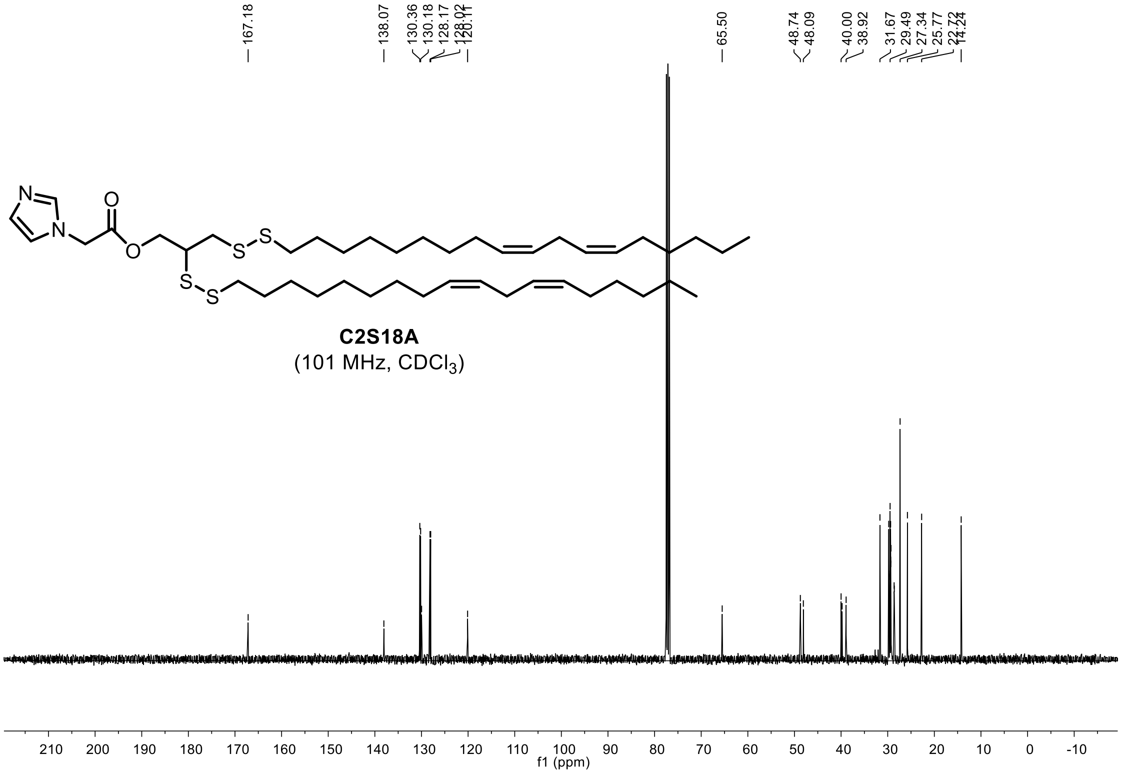

167.18
138.07
130.36
130.18
128.17
128.02
120.11
65.50
48.74
48.09
40.00
38.92
31.67
29.49
27.34
25.77
22.72
14.24
C2S18A
(101 MHz, $CDCl_3$)
f1 (ppm)

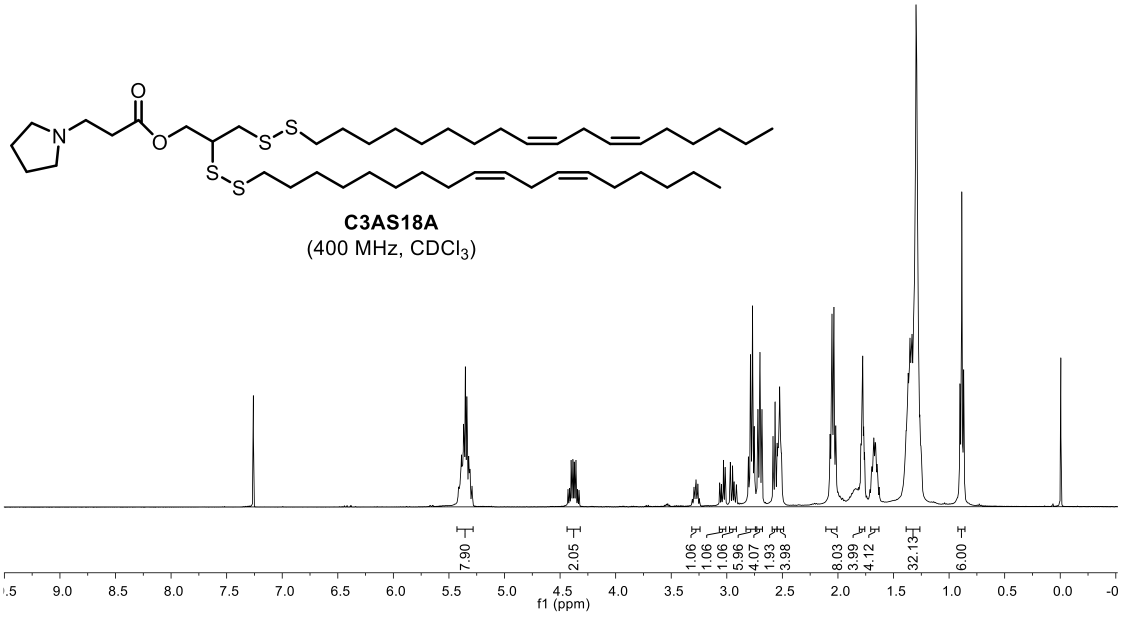
C3AS18A
(400 MHz, $CDCl_3$)
7.90
2.05
1.06
1.06
1.06
5.96
4.07
1.93
3.98
8.03
3.99
4.12
32.13
6.00
f1 (ppm)

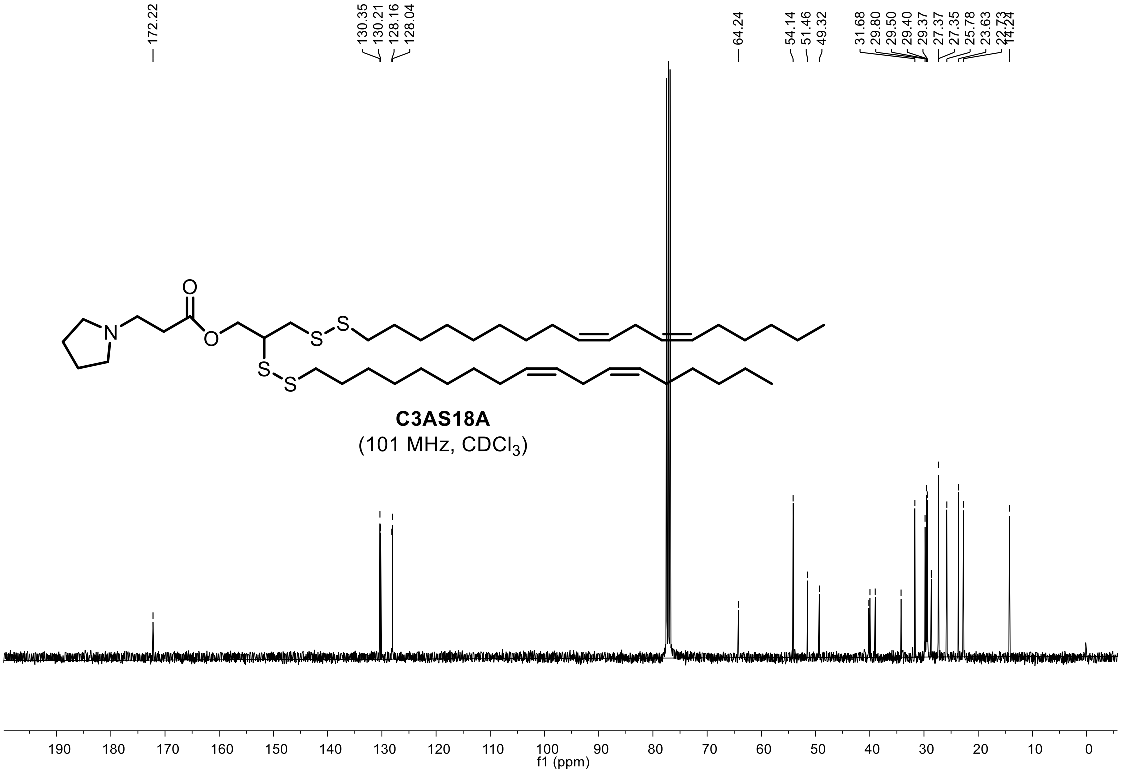
172.22
130.35
130.21
128.16
128.04
64.24
54.14
51.46
49.32
31.68
29.80
29.50
29.40
29.37
27.37
27.35
25.78
23.63
22.73
14.24
C3AS18A
(101 MHz, $CDCl_3$)
f1 (ppm)

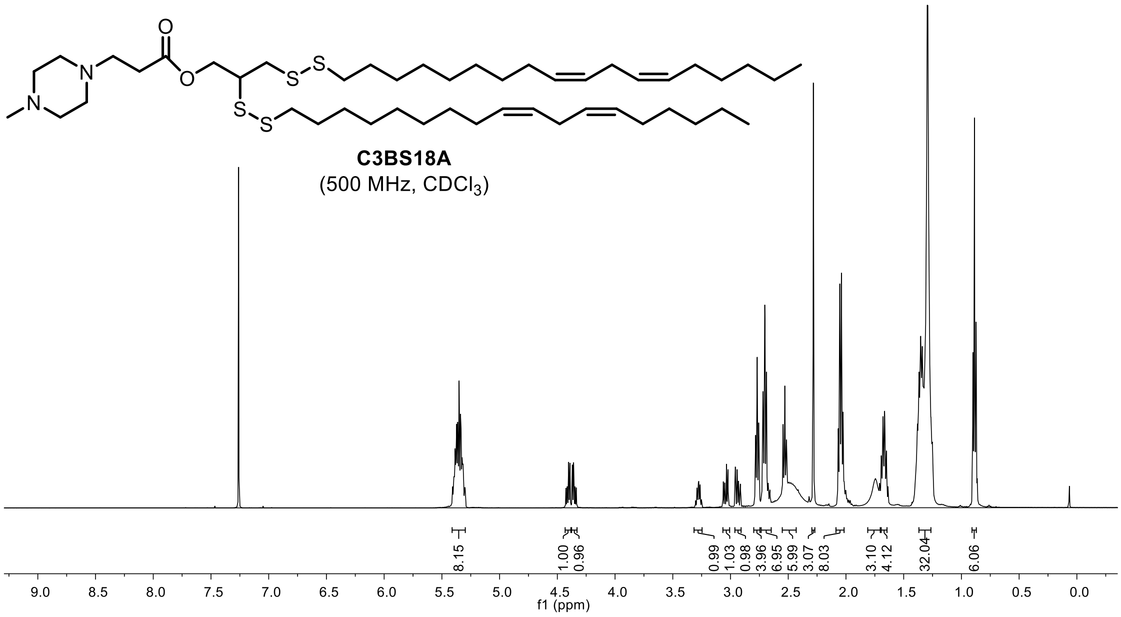
C3BS18A
(500 MHz, $CDCl_3$)
8.15
1.00
0.96
0.99
1.03
0.98
3.96
6.95
5.99
3.07
8.03
3.10
4.12
32.04
6.06
f1 (ppm)

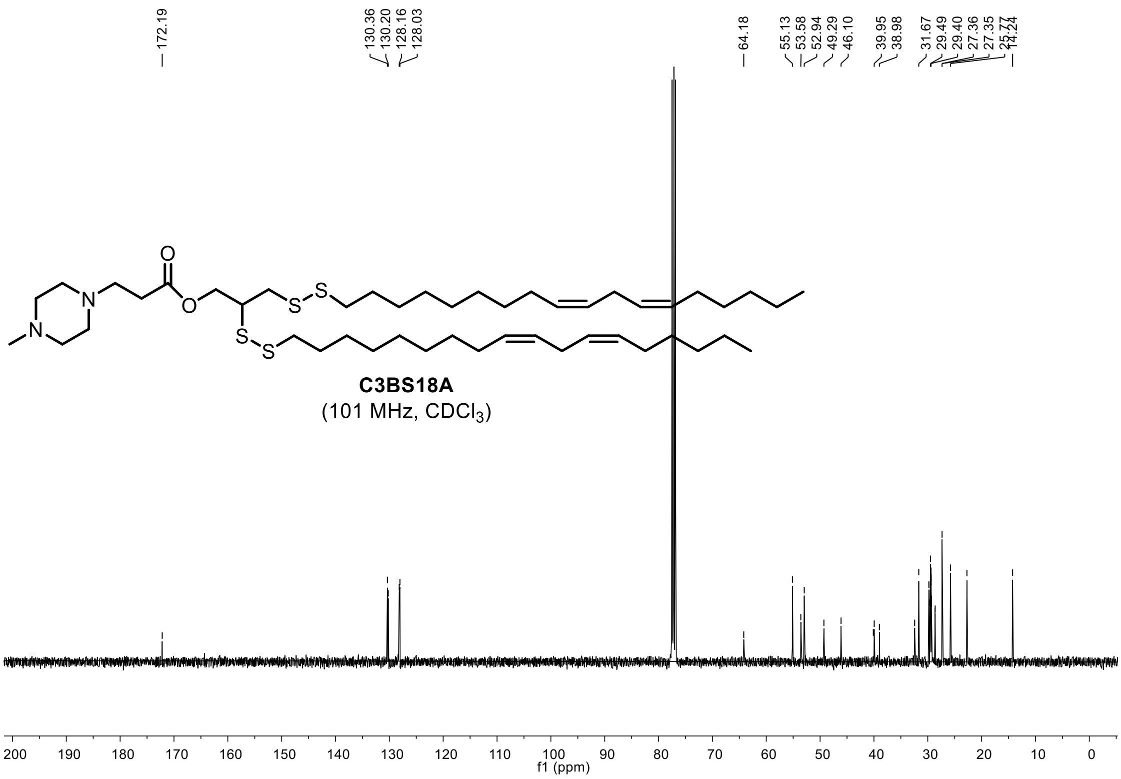
172.19
130.36
130.20
128.16
128.03
64.18
55.13
53.58
52.94
49.29
46.10
39.95
38.98
31.67
29.49
29.40
27.36
27.35
C3BS18A
(101 MHz, $CDCl_3$)
f1 (ppm)

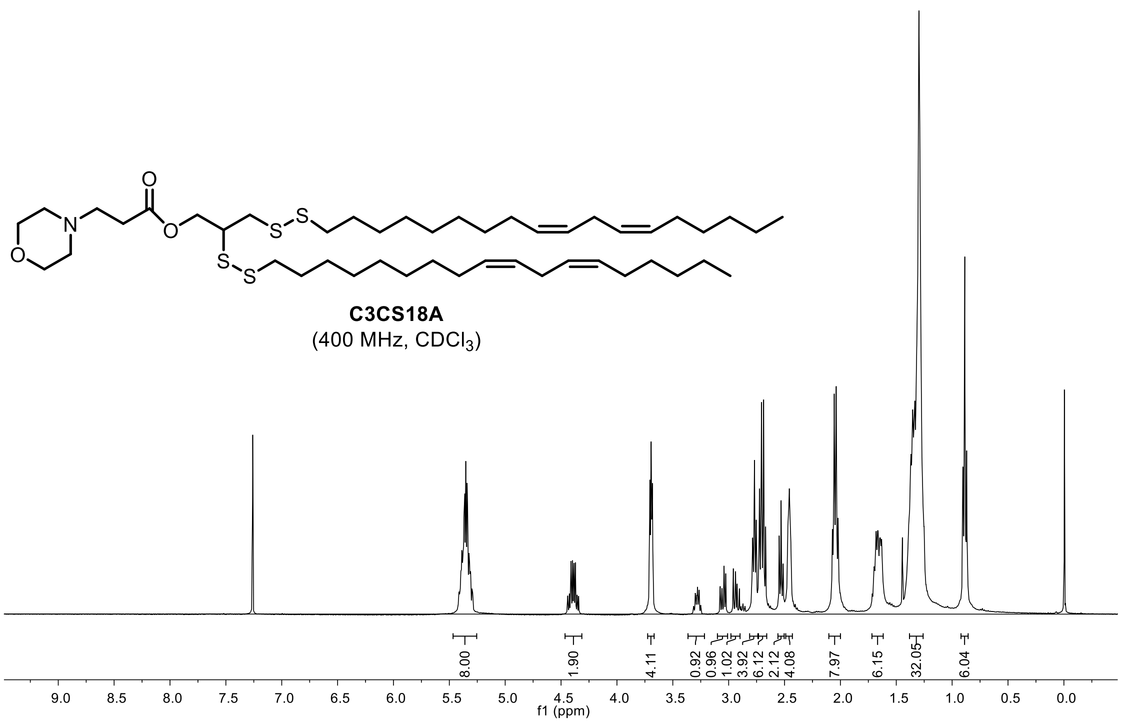
C3CS18A
(400 MHz, $CDCl_3$)
8.00
1.90
4.11
0.92
0.96
1.02
3.92
6.12
2.12
4.08
7.97
6.15
32.05
6.04
f1 (ppm)

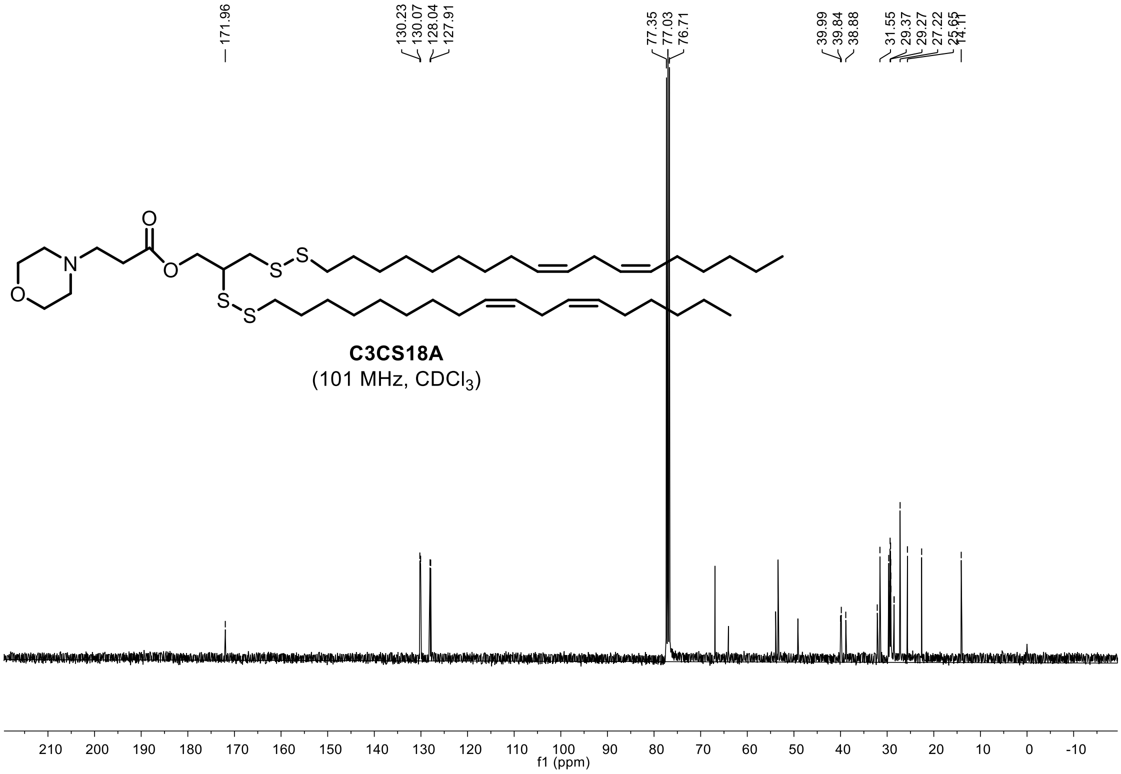
171.96
130.23
130.07
128.04
127.91
77.35
77.03
76.71
39.99
39.84
38.88
31.55
29.37
29.27
27.22
25.65
14.11
C3CS18A
(101 MHz, $CDCl_3$)
f1 (ppm)

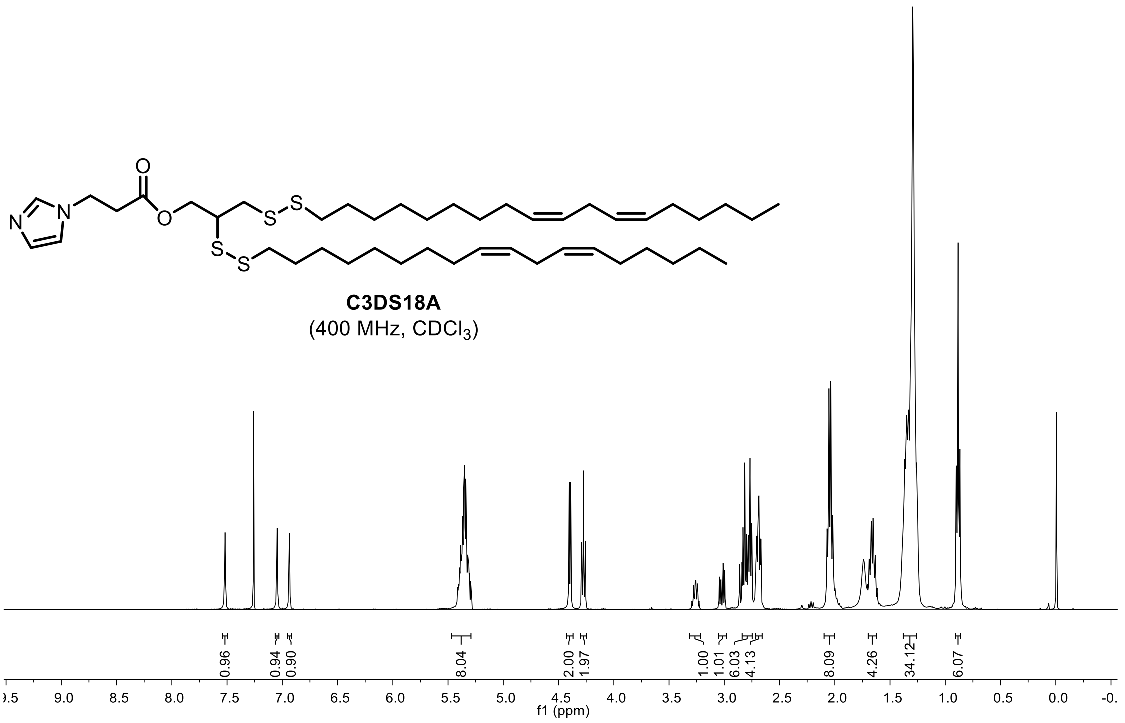
C3DS18A
(400 MHz, CDCl$_3$)
f1 (ppm)

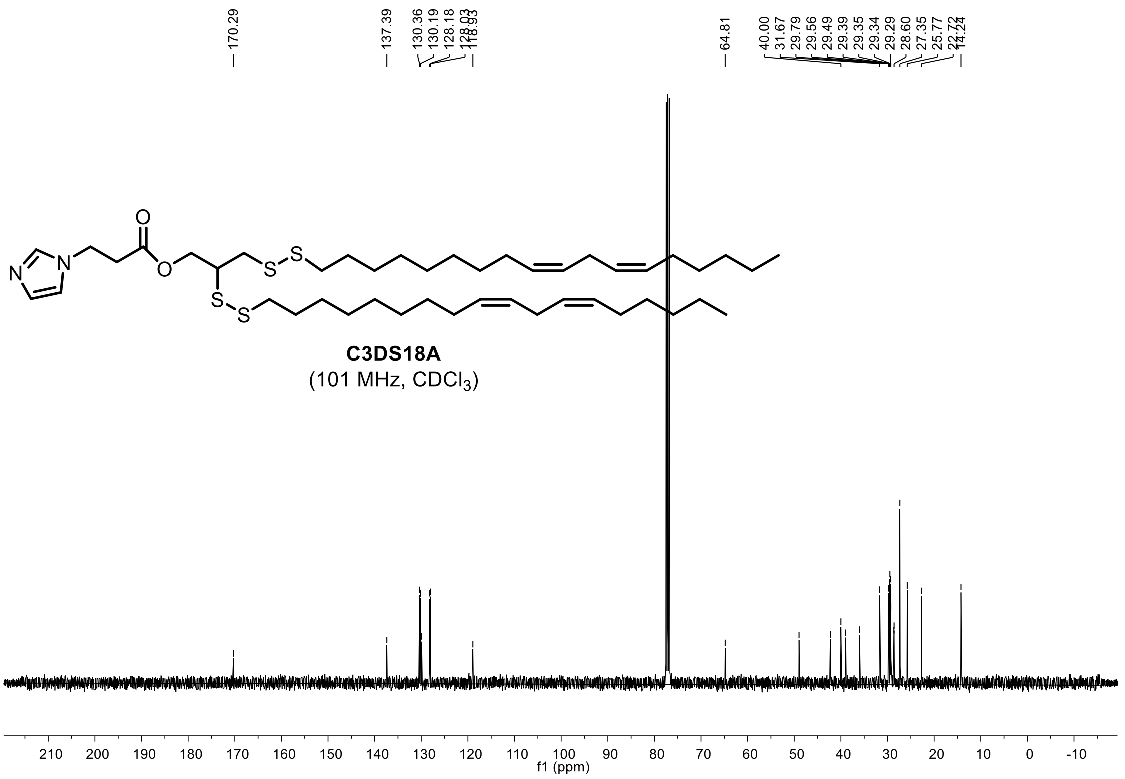
C3DS18A
(101 MHz, CDCl$_3$)
f1 (ppm)
